# Relative binding free energy calculations with transformato: A molecular dynamics engine-independent tool

**DOI:** 10.3389/fmolb.2022.954638

**Published:** 2022-09-06

**Authors:** Johannes Karwounopoulos, Marcus Wieder, Stefan Boresch

**Affiliations:** ^1^ Faculty of Chemistry, Institute of Computational Biological Chemistry, University of Vienna, Vienna, Austria; ^2^ Vienna Doctoral School of Chemistry (DoSChem), University of Vienna, Vienna, Austria; ^3^ Department of Pharmaceutical Sciences, Faculty of Life Sciences, University of Vienna, Vienna, Austria

**Keywords:** free energy, molecular dynamics simulation, binding affinity, automated setup, open source, python (programming language)

## Abstract

We present the software package transformato for the setup of large-scale relative binding free energy calculations. Transformato is written in Python as an open source project (https://github.com/wiederm/transformato); in contrast to comparable tools, it is not closely tied to a particular molecular dynamics engine to carry out the underlying simulations. Instead of alchemically transforming a ligand *L*_1_ directly into another *L*_2_, the two ligands are mutated to a common core. Thus, while dummy atoms are required at intermediate states, in particular at the common core state, none are present at the physical endstates. To validate the method, we calculated 76 relative binding free energy differences $\Delta \Delta G_{L_{1}\to L_{2}}^{bind}$ for five protein–ligand systems. The overall root mean squared error to experimental binding free energies is 1.17 kcal/mol with a Pearson correlation coefficient of 0.73. For selected cases, we checked that the relative binding free energy differences between pairs of ligands do not depend on the choice of the intermediate common core structure. Additionally, we report results with and without hydrogen mass reweighting. The code currently supports OpenMM, CHARMM, and CHARMM/OpenMM directly. Since the program logic to choose and construct alchemical transformation paths is separated from the generation of input and topology/parameter files, extending transformato to support additional molecular dynamics engines is straightforward.

## 1 Introduction

The accurate prediction of relative protein–ligand binding affinities is one of the major tasks in computer-aided drug design projects, especially during lead optimization. A group of methods often referred to as alchemical free energy simulations has become a versatile tool in this area, e.g., (Deflorian et al., 2020; Majellaro et al., 2020; Mortier et al., 2020; Schindler et al., 2020; Zhang C.-H. et al., 2021). While relative binding free energy (RBFE) simulations have been successful in reproducing and predicting experimental results, their application in drug design projects is still far from routine. They require significant computing resources and are comparatively slow; setting up the simulations and analysis procedures is difficult and tedious, even for experts. To make their utilization easier, several front ends to biomolecular simulation packages have been developed to help set up RBFE simulations (Seeliger and de Groot, 2010; Gapsys et al., 2015; Loeffler et al., 2015; Wang et al., 2015; Zhang H. et al., 2021; Petrov, 2021). Here, we present a related tool, transformato, which, in contrast to most other tools, is not dependent on a particular simulation program. Transformato is a Python package that automates the setup and calculation of relative solvation and binding free energy calculations using the common core/serial-atom-insertion (CC/SAI) approach (Wieder et al., 2022). The CC/SAI approach avoids the need for special-purpose code (mixing of energy terms, soft-core potentials, etc.), making it possible to carry out RBFE calculations with standard molecular dynamics (MD) engines. Specifically, transformato is not restricted to a specific MD program; the code currently supports CHARMM and OpenMM.

### 1.1 Introduction to transformato—the common core/serial-atom-insertion approach

**FIGURE 1:**
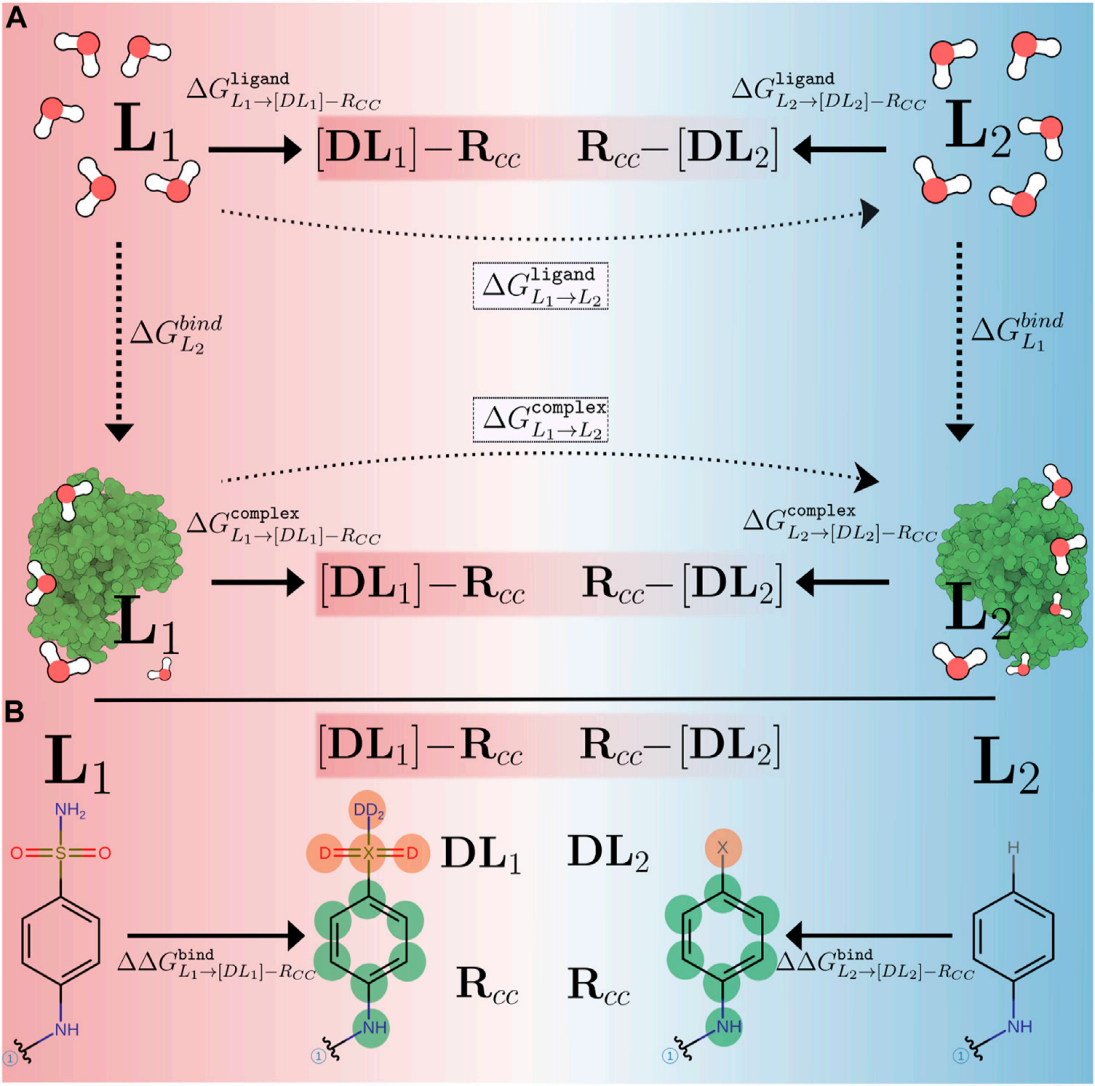
**(A)** Comparison of alchemical paths used in traditional setups (dotted, horizontal arrows) and in the CC/SAI approach (thick, horizontal arrows) implemented in transformato to compute relative binding free energy differences. Free energies are calculated relative to non-physical intermediate states, the common core [DL_*i*_]-R_CC_ (*i* = 1, 2), connecting the two ligands (L_1_, L_2_). Here [DL_*i*_] represents the atoms of each ligand not in the CC region and R_CC_ indicates the CC region itself. **(B)** The mutation path for calculating $\Delta \Delta G_{L_{1}\to L_{2}}^{bind}$ between a pair of ligands taken from the CDK2 dataset. The atoms that differ between the two ligands and, thus, are not present in the CC, are highlighted in color; these are transformed into dummy atoms (reddish circles). The common core is indicated by the green circles.

Figure 1 illustrates the traditional and the CC/SAI approach, implemented in transformato, to compute the RBFE difference between two ligands L_1_ and L_2_. In both cases, one avoids the direct calculation of the binding free energies ${\Delta G^{bind}}_{L_{1}}$ and ${\Delta G^{bind}}_{L_{2}}$ (vertical dashed arrows in Figure 1A) and considers instead the alchemical transformation of L_1_ into L_2_, which is carried out for the free ligand in water $\left(\Delta G_{L_{1}\to L_{2}}^{\mathrm{ligand}}\right)$ and for the ligand in complex with the receptor $\left(\Delta G_{L_{1}\to L_{2}}^{\mathrm{complex}}\right)$ . Since the free energy is a state function, the quantity of interest, the RBFE difference $\Delta \Delta G_{L_{1}\to L_{2}}^{bind}={\Delta G^{bind}}_{L_{2}}-{\Delta G^{bind}}_{L_{1}}$ , can also be calculated as $\Delta \Delta G_{L_{1}\to L_{2}}^{\mathrm{bind}}=\Delta G_{L_{1}\to L_{2}}^{\mathrm{complex}}-\Delta G_{L_{1}\to L_{2}}^{\mathrm{ligand}}$ (Tembre and McCammon, 1984).

In the traditional approach, indicated by the dotted arrows in Figure 1A, this is done in a single transformation. The ligand *L*_1_ is “morphed” into *L*_2_ by gradually scaling the force field parameters associated with all atoms that are different in the two ligands along a non-physical coordinate—the so-called coupling parameter *λ*. For each environment (the ligand in aqueous solution, protein–ligand complex in aqueous solution), several simulations, typically 10–20, are performed at different values of *λ*. Such transformations can be set up using either the single topology or dual topology paradigm (Pearlman, 1994). One important practical detail is that the number of atoms must not change. Since in most cases the two ligands do not consist of exactly the same number of atoms, so-called dummy atoms need to be introduced along the alchemical paths (single topology). In dual topology, all groups are present simultaneously and interactions with the remainder of the system are turned on/off as needed. Dummy atoms (single topology), as well as atoms in non-interacting groups (dual topology), are particles that stay connected to the physical molecule by bonded terms, but do not take part in any Lennard-Jones (LJ) or Coulomb interactions (Mey et al., 2020; Fleck et al., 2021). One can show that the presence of dummy atoms has no influence on double free energy differences, such as the RBFE differences considered here, but some care concerning their treatment is required (Fleck et al., 2021). The correct handling of dummy atoms or non-interacting groups in an automated manner is one of the challenges in large-scale FES.

In the common core (CC) approach, rather than alchemically transforming *L*_1_ into *L*_2_ directly, one defines a suitable common substructure present in both ligands, which we refer to as the CC. It is not necessary that the CC corresponds to a physical molecule (Wieder et al., 2022). The atoms of a ligand that are not part of the CC, are mutated to dummy atoms (hereafter, referred to as *non-CC atoms*). Starting from each of the physical endstates L = {L_1_, L_2_}, we compute the free energy difference between the ligand and the common core [DL]-R_CC_, as indicated in Figure 1A. Here [DL] indicates the non-CC atoms transformed into dummy atoms at the CC state, and R_*CC*_ denotes the interacting atoms belonging to the CC. This is done for the free ligand solvated in water $\Delta G_{L\to \left[DL\right]-R_{CC}}^{\mathrm{ligand}}$ , as well as for the protein–ligand complex $\Delta G_{L\to \left[DL\right]-R_{CC}}^{\mathrm{complex}}$ . Thus, for each of the ligands, we can compute a RBFE difference with respect to a CC:

$$\Delta \Delta G_{L\to \left[DL\right]-R_{CC}}^{\mathrm{bind}}=\Delta G_{L\to \left[DL\right]-R_{CC}}^{\mathrm{complex}}-\Delta G_{L\to \left[DL\right]-R_{CC}}^{\mathrm{ligand}}$$ (1)

When mutating the non-CC atoms of two ligands to dummy atoms, the CCs reached from L_1_ and L_2_, respectively, are not necessarily identical. Most importantly, if the physical endstates L_1_ and L_2_ consist of different numbers of atoms, then the corresponding CCs will contain different numbers of dummy atoms. However, if these dummy atoms are treated correctly, they do *not* influence double free energy differences (Fleck et al., 2021); in other words, $\Delta \Delta G_{L\to \left[DL\right]-R_{CC}}^{\mathrm{bind}}$ of Eq. 1 is not affected by the number of dummy atoms present at the CC endpoint. Provided that the remaining differences between the CCs, if any, are accounted for (see below and Section 2.4 for further details), the RBFE difference $\Delta \Delta G_{L_{1}\to L_{2}}^{\mathrm{bind}}$ between the two ligands in the CC framework is obtained as:

$$\Delta \Delta G_{L_{1}\to L_{2}}^{\mathrm{bind}}=\Delta \Delta G_{L_{1}\to \left[DL_{1}\right]-R_{CC}}^{\mathrm{bind}}-\Delta \Delta G_{L_{2}\to \left[DL_{2}\right]-R_{CC}}^{\mathrm{bind}}$$ (2)

The approach just outlined can of course be extended to more than two ligands. If a suitable CC is chosen, then all pairwise RBFE differences between *N* ligands can be obtained from just *N* calculations of $\Delta \Delta G_{L_{i}\to \left[DL_{i}\right]-R_{CC}}^{\mathrm{bind}}$ (cf. Eq. 1).

In Figure 1B we show a specific alchemical transformation studied in this work to illustrate these general considerations. The two ligands (L_1_, L_2_) are shown on the left and right-hand side, respectively. The black (non-colored) parts of the structures represent the CCs. The regions that are different between the two ligands are drawn in color, with their atoms labeled in boldface. For L_1_, there are six non-CC atoms that need to be transformed into dummy atoms, while for L_2_, only a single hydrogen atom needs to be mutated. The electrostatic and LJ interactions of the non-CC atoms with the environment (solvent and/or protein) and the atoms belonging to the CC are turned off using the serial atom insertion (SAI) approach (Boresch and Bruckner, 2011; Wieder et al., 2022). The detailed methodology and sequence of steps used are described in full detail in *Methods* (Section 2.4). Although the different number of dummy atoms has no influence on $\Delta \Delta G_{L_{1}\to L_{2}}^{\mathrm{bind}}$ as computed according to Eq. 2 (cf. above), some other force field parameters of the 2 CCs are different. In order to make the CCs identical and to close the thermodynamic cycle, additional modifications need to be applied to one of the ligands, specifically its CC, during a final stage. In the example shown in Figure 1B, the partial charges of the atoms in the phenyl ring, to which the dummy atom(s) are connected, are slightly different since the charge distribution in the physical ligands differs. A more detailed description of the modifications needed to ensure that the CCs are identical is given in Section 2.4.

### 1.2 Goals of this work

We recently used transformato to compute relative solvation free energy differences and demonstrated that one can obtain high accuracy and precision with the CC/SAI approach (Wieder et al., 2022). In this work we report first results of RBFE calculations obtained with transformato as the setup tool. Specifically, we carried out 76 pairwise mutations from well established datasets (Homeyer and Gohlke, 2013; Wang et al., 2015; Gapsys et al., 2020) and compare our results both to the experimental reference data, as well as to the earlier computational results obtained by other groups. We either directly compare ΔΔ*G* ^*bind*^ values for pairs of ligands, or, by using one of the experimental binding affinities, express our results as absolute binding free energies, as was done in some of the reference studies (Wang et al., 2015; Hu et al., 2016; Song et al., 2019). In addition to the overall performance of transformato, we also investigated a number of additional aspects. First, for selected ligand pairs we computed RBFE differences using different CCs, thus proving the self-consistency of the CC/SAI approach. Second, while we used OpenMM (Eastman et al., 2017) as the underlying MD program in all of our calculations, we repeated a subset of alchemical transformations with CHARMM as the computational backend (Brooks et al., 2009). Third, we achieve computational efficiency by the consequent use of hydrogen mass reweighting (HMR) (Hopkins et al., 2015) in the underlying MD simulations. The use of HMR for RBFE simulations was recently studied and discussed by Zhang H. et al. (2021). To validate that HMR can be safely used in FES, we report results obtained with and without HMR for the same subset of alchemical transformations used in the OpenMM to CHARMM comparison. Finally, while we use the CHARMM family of force fields (see *Methods*), in one case we explored the effect of using different charge models.

## 2 Methods

### 2.1 Choice of datasets

To validate the CC/SAI approach as implemented in transformato for RBFE calculations, we selected five benchmark applications for which experimental binding affinities are known and that have been studied extensively in previous work. Three of these were taken from Wang et al. (2015), i.e., JNK1 (Supplementary Figure S1), CDK2 (Supplementary Figure S2), and TYK2 (Supplementary Figure S3). In addition, we investigated the FXa system (Supplementary Figure S4), first studied by Homeyer and Gohlke (2013) and reevaluated by Hu et al. (2016). Finally, we chose a set of inhibitors of galactin-3 (GAL3, Supplementary Figure S5) studied by Gapsys et al. (2020) and earlier by Manzoni and Ryde (2018). For these five protein–ligand datasets, we compare our results to the experimentally determined Δ*G* ^*bind*^ values, as well as to calculated values reported in the respective literature. For the JNK1, FXa and GAL3 datasets we calculated $\Delta \Delta G_{L_{1}\to L_{2}}^{\mathrm{bind}}$ for the ligand pairs used in the original studies (Wang et al., 2015; Hu et al., 2016; Gapsys et al., 2020). For the CDK2 and TYK2 dataset, we employed a different approach and mutated each ligand to a CC resembling the respective smallest ligand. Thus, for JNK1, FXa, GAL3 multiple CCs were required to compute the specific RBFE differences, whereas in the second case (CDK2, TYK2) only a single CC was needed. All comparisons to the study by Gapsys et al. (2020) are based on their results obtained with the CHARMM Generalized force field (CGenFF), the force field used in this work (see below); we indicate this by the abbreviation pmx/CGenFF. An overview of the systems studied and the previous computational results we compare to is shown in Table 1.

**TABLE 1:** Overview of systems and previous computational studies.

System	PDB ID	No. of	Program	Force field (+ charge assignment)		References
		ligands		ligand	protein	
JNK1	2GMX	21	FEP+	OPLS2.1	OPLS2.1	Wang et al. (2015)
			AMBER-TI	RESP charges/GAFF 1.8	ff14SB	Song et al. (2019)
			pmx/CGenFF	CGenFF v3.0.1 (v4.1)	CHARMM36m	Gapsys et al. (2020)
FXa	2RA0	11	AMBER-TI	AM1-BCC/GAFF	ff12SB	Hu et al. (2016)
GAL3	5E89	8	pmx/CGenFF	CGenFF v3.0.1 (v4.1)	CHARMM36m	Gapsys et al. (2020)
CDK2	1H1Q	14	FEP+	OPLS2.1	OPLS2.1	Wang et al. (2015)
			AMBER-TI	RESP charges/GAFF 1.8	ff14SB	He et al. (2020)
			pmx/CGenFF	CGenFF v3.0.1 (v4.1)	CHARMM36m	Gapsys et al. (2020)
TYK2	4GIH	15	FEP+	OPLS2.1	OPLS2.1	Wang et al. (2015)
			AMBER-TI	RESP charges/GAFF 1.8	ff14SB	Song et al. (2019)
			pmx/CGenFF	CGenFF v3.0.1 (v4.1)	CHARMM36m	Gapsys et al. (2020)

### 2.2 Dataset preparation

For CDK2, TYK2, and JNK1 structural information for the protein and the ligands was taken from the supporting information of Wang et al. (2015). Similarly, protein structure files for the GAL3 dataset were obtained from Gapsys et al. (2020). In both cases we used Maestro (Release 2021-3, Schrödinger, LLC, New York, NY, 2021) to prepare starting coordinates for the protein–ligand complexes.

The protein–ligand structures for the FXa system were generated as described by Homeyer and Gohlke (2013) by starting from the high resolution crystal structure of the Factor Xa-L51a complex (PDB code: 2RA0). The ligand present in this PDB entry (l51a) served as the template to model structures for the other ligands of the series (l51b–l51k). We used the protonation state of the ligands suggested by Hu et al. (2016). For each modelled FXa protein–ligand structure we performed a short minimization of the ligand in the binding site with the built-in minimizer of Maestro.

No attempts were made to optimize the protonation state of the proteins. All Asp, Glu, Lys and Arg residues were assumed to be in their charged state, and all histidines were set to neutral with the proton on the *δ*-nitrogen.

### 2.3 CHARMM-GUI preparation

**FIGURE 2:**
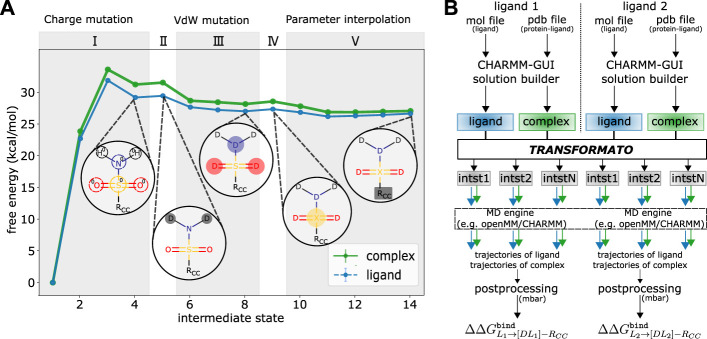
**(A)** Details of the mutation path from *L*_1_ shown in Figure 1B) to its CC to compute $\Delta \Delta G_{L_{1}\to \left[DL_{1}\right]-R_{CC}}^{\mathrm{bind}}$ : (I) electrostatic interactions of the non-CC atoms are scaled to zero (three steps; step 1 in the diagram is the native, fully interacting ligand), (II) LJ interactions of the non-CC hydrogen atoms are turned off (one step), (III) LJ interactions of the three non-CC heavy atoms are turned off on an atom-by-atom basis (three steps), (IV) the last non-CC heavy atom is changed to the “junction” atom type X (one step), (V) the CC reached from *L*_1_ $\left(R_{cc_{1}}\right)$ is adjusted to the one reached from *L*_2_ (cf. Figure 1B), in five steps. **(B)** Overview of the workflow when computing free energy differences with transformato.

The PDB files of the ligand and of the protein–ligand complexes were uploaded to the Solution Builder functionality of CHARMM-GUI (Jo et al., 2008; Lee et al., 2016) to create input files for the ligand in water (“ligand”) and for the ligand bound to the protein (“complex”). This is the first step of the workflow depicted in Figure 2B. All systems were made electrically neutral by adding a suitable number of potassium and chloride ions (JNK1, GAL3, CDK2, and TYK2) or calcium and chloride ions for FXa, see Hu et al. (2016). Ligand parameters were generated with CGenFF (v2.5) (Vanommeslaeghe et al., 2010; Vanommeslaeghe et al., 2012; Vanommeslaeghe and MacKerell, 2012; Yu et al., 2012; Gutiérrez et al., 2016). The CHARMM36m force field (Huang et al., 2016) was used for the proteins, and TIP3P was employed as the water model (Jorgensen et al., 1983). Furthermore, during setup with CHARMM-GUI we prepared the systems for HMR. Following Hopkins et al. (2015), hydrogen masses were multiplied by a factor of 3 while the masses of the heavy atoms they are bound to were lowered accordingly to maintain a constant molecular mass. This allowed us to use a time-step of 4 fs (all bond lengths to hydrogens constrained), thus considerably lowering the computational cost of the subsequent MD simulations. A subset of mutations was repeated without HMR; here a time-step of 1 fs was used and the ligands were fully flexible.

At the end of the preparatory steps with CHARMM-GUI just outlined, one obtains two folders for each protein–ligand system; one for the ligand, one for the protein–complex. Each contains all input and parameter files to run MD simulations of the respective system. Each set of initial coordinates was equilibrated for 125 ps under NVT conditions with weak position restraints applied to the heavy atoms of the protein [force constant of 400 kJ/mol/nm^2^ on the protein backbone atoms and 40 kJ/mol/nm^2^ on atoms in the protein side-chain; these are the CHARMM-GUI recommended defaults (Lee et al., 2016)]. These inputs and coordinate files for the native systems serve as the basis from which transformato constructs the mutations paths between pairs of ligands and input files for the simulation of intermediate states (see Figure 2B).

### 2.4 Practical aspects of transformato

For the mutation of *L*_1_ to *L*_2_, transformato first identifies the maximum common substructure, which forms the basis for the CCs [*DL*_*i*_]-*R*_*CC*_ of Figure 1A. For each ligand transformato constructs an alchemical path along which the atoms not belonging to the maximum common substructure are mutated to dummy atoms using the SAI approach. At this point, however, the CCs reached from *L*_1_ and *L*_2_, respectively, are not necessarily identical (cf. the *Introduction*). Thus, for one of the ligands transformato generates the required, additional steps for the transformation $\left[DL_{1}\right]-R_{CC_{1}}\to \left[DL_{1}\right]-R_{CC_{2}}$ . For each intermediate step along the alchemical path *L*_*i*_ → [*DL*_*i*_]-*R*_*CC*_ (*i* = 1, 2), transformato creates all necessary files for running the MD simulations; thus, each of these states can be sampled independently. During the MD simulations coordinates are saved to disk; from these trajectories the energies at the respective other states, needed by the (multi-state) Bennett acceptance ratio method (MBAR) (Shirts and Chodera, 2008), are extracted in a post-processing step (see below).

We illustrate the above by describing in detail the steps required to transform *L*_1_ shown in Figure 1B to its CC. All steps are illustrated in Figure 2A. The exact number and sequence of intermediate steps needed for this particular transformation is depicted; in general, it depends on the details of the mutation (see below). During the first stage (I) the electrostatic interaction of the non-CC atoms are scaled linearly to zero. In all transformations considered in this work, three intermediate states were used. Based on our experience, depending on the polarity and number of atoms, up to five intermediates may be necessary. To ensure that the overall charge of the system remains unchanged as the charges of the non-CC atoms are turned off, a compensating partial charge is added to the real atom of the CC to which the non-CC atoms are connected. While this stage corresponds to a linear dependence of the partial charges on a continuous coupling parameter *λ* as in traditional approaches, transformato, nevertheless, generates self-contained input files for each intermediate state.

Next, the LJ interactions of the non-CC atoms are turned off using the SAI approach (Boresch and Bruckner, 2011). First (stage II), the LJ interactions of all non-CC hydrogen atoms are turned off. in a single step. For this stage, a single step is always sufficient, regardless of the number of hydrogen atoms. Then (stage III), the LJ interactions of the non-CC heavy atoms are turned off atom-by-atom. In this work, cf. Figure 2, in each step the LJ interaction of only a single heavy atom was turned off. We strongly advise against turning off the interactions of more than two heavy atoms simultaneously to ensure sufficient overlap between neighboring states. The treatment of the “last” non-CC heavy atom (stage IV), i.e., the atom directly connected to an atom in the CC region, is special; cf. Wieder et al. (2022). Rather than mutating it to a dummy atom (no LJ interactions), this “junction” atom X retains some LJ-interactions (note, though, that its partial charge is zero). By means of this junction atom, all dummy atoms are attached to the CC via a “terminal junction.” Fleck et al. (2021) demonstrated that this guarantees that any contributions from dummy atoms cancel from the double free energy differences of interest. Transformato ensures that in any transformation from *L*_1_ to *L*_2_, the junction atoms X of the CCs have identical parameters. Since state (IV) is only a change in LJ interactions, one intermediate step is always sufficient.

Finally (V), the parameters of the CC of *L*_1_ are modified so that they become identical to the parameters of the CC reached from *L*_2_. In the specific example (see Figure 1B), two types of parameter changes are required: 1) since the charge distributions in the phenyl rings of the two ligands are not the same in the real molecules, these need to be made identical at the CC endpoint. 2) The parameters of the bond between the junction atom X and the physical CC-atom it is connected to have to be made identical. At the end of stage (IV), in *L*_1_ the C-X bond is described by the parameters of the C-S bond of the native ligand, whereas in *L*_2_, the parameters are those of a C-H bond. During stage V, the partial charges and bonded parameters involving X of the CC reached from *L*_1_ are simultaneously modified linearly in five steps (see Figure 2A). The number of intermediate states is rather system dependent; the most critical factor affecting convergence is the change of the equilibrium bond-length from the last physical atom to the junction atom X; the change in bond-length per step should be ≤0.125 Å. During stage (V) there is a strict one to one correspondence between interacting atoms; all alchemical changes are carried out in the single topology paradigm by parameter mixing. As during all earlier stages, transformato writes a complete set of input/parameter files for each of these intermediate states.

More extensive differences between the CCs of *L*_1_ and *L*_2_ are permitted. The main requirement for the CCs, as used by transformato, is that there needs to be an unambiguous correspondence between each of the atoms. If, e.g., the common substructure search is based on element identity, then two atoms are considered to correspond to each other if they have the same element, even if they have different hybridization states or atom types. In this case, additional parameter changes need to be applied during stage (V) that are essential to ensure the validity of Eq. 2. For a given ligand pair, stages (I)–(IV) need to be carried out for each of the ligands, whereas stage (V) is only required for one of them. In our specific example (Figure 1B), the CC reached from *L*_2_ is considered the endstate that must be also reached starting from *L*_1_ to close the thermodynamic cycle.

### 2.5 Details of the MD simulations

As shown in Figure 2B and described in the previous section, transformato generates all necessary input files to perform MD simulations for each intermediate state, both for the ligand in solution, as well as for the full protein–ligand complex. All simulations were performed using the OpenMM software package (version 7.5) (Eastman et al., 2017). A subset of the mutations was also carried out using CHARMM/OpenMM (version c47a1) (Brooks et al., 2009). For each intermediate state a Langevin dynamics simulation of 5 ns length was carried out at 303.15 K; the friction coefficient was set to 1/ps. All simulations, except the short equilibration runs with position restraints described earlier, were carried out under constant pressure conditions. The pressure was controlled using a Monte Carlo barostat (Chow and Ferguson, 1995; Åqvist et al., 2004). Waters were kept rigid throughout the simulation using the SETTLE (Miyamoto and Kollman, 1992) (OpenMM) or SHAKE algorithm (Ryckaert et al., 1977) (CHARMM/OpenMM). Coulomb interactions were calculated using the particle-mesh Ewald (PME) method (Essmann et al., 1995). LJ interactions were switched smoothly to zero between 10 Å and 12 Å using the CHARMM force-switching function (Steinbach and Brooks, 1994). Production runs for each of the intermediate states were started from the respective restart file generated during the equilibration (see Section 2.3). Prior to each production run, the coordinates were optimized using the L-BFGS algorithm in OpenMM or the steepest descent and adopted basis Newton-Raphson minimizer in CHARMM/OpenMM. Simulations of each state were repeated three times with different random initial velocities.

### 2.6 Calculation of relative binding free energy differences $\left(\Delta \Delta G_{L_{1}\to L_{2}}^{\mathrm{bind}}\right)$

During each of the MD simulations described in the previous section, coordinates were written to disk every 250 steps. Using HMR and a time-step of 4 fs, each trajectory, therefore, contained 5,000 frames. The first 25% of each trajectory were considered as equilibration and discarded; the remaining coordinate sets were used to recompute the energies at all other intermediate states. All scripts for this post-processing are generated by transformato, which then invokes the MBAR functionality of pymbar (Shirts and Chodera, 2008) to compute the free energy differences $\Delta G_{L_{i}\to \left[DL_{i}\right]-R_{CC}}^{\mathrm{ligand}}$ and $\Delta G_{L_{i}\to \left[DL_{i}\right]-R_{CC}}^{\mathrm{complex}}$ (*i* = 1, 2). For each intermediate state *λ* and each configuration sample x, the reduced potential u (x,*λ*) was computed to form the N × K matrix of inputs, where N is the number of snapshots used and K is the number of alchemical states *λ*_*k*_ for a given transformation. Finally, for both ligands *L*_1_ and *L*_2_ we obtained $\Delta \Delta G_{L_{i}\to \left[DL_{i}\right]-R_{CC}}^{\mathrm{bind}}$ , from which we computed the RBFE difference $\Delta \Delta G_{L_{1}\to L_{2}}^{\mathrm{bind}}$ according to Eq. 2. Since each set of simulations was repeated three times, using different independent initial velocities (cf. above), we obtained three statistically independent free energy differences. We used these to estimate the statistical error; when the directly computed free energy differences were combined/processed further (see below), Gaussian error propagation was used.

### 2.7 Expressing the results as absolute binding free energies (Δ*G* ^*bind*^)

To validate a tool such as transformato, one needs to compare both to other computational methods, as well as to experimental data. Transformato leads directly to RBFE differences $\Delta \Delta G_{L_{1}\to L_{2}}^{\mathrm{bind}}$ . These can be compared across different computational methods easily only for identical pairs of ligands. As described in Section 2.1, for three model applications (JNK1, FXa and GAL3) we computed exactly the same RBFE differences for which results were reported by others.

Comparison of the results obtained with different methods and, in particular, comparison to experimental data, is much easier using absolute binding free energy differences Δ*G* ^*bind*^. For this reason, in the past several authors have expressed results of RBFE calculations in terms of Δ*G* ^*bind*^ (Wang et al., 2015; Song et al., 2019; He et al., 2020). There are several options for post-processing $\Delta \Delta G_{L_{1}\to L_{2}}^{\mathrm{bind}}$ , such as the “cycle closure” (Wang et al., 2015) or “centered RMSE” (Gathiaka et al., 2016) approaches, which attempt to improve the overall results and make the corresponding RMSE and MAE lower (Song et al., 2019). In this work we used the simplest possible approach: for each system we chose one ligand *L*_1_ and considered its experimental $\Delta G_{L_{1}}^{\mathrm{bind}}$ as the reference value. With the help of this reference ligand/value, we computed the “absolute” binding free energies $\Delta G_{i}^{\mathrm{bind}}$ (*i* = 2, … *n*, with *n* being the total number of ligands considered for this system) for all other ligands according to $\Delta G_{L_{i}}^{\mathrm{bind}}=\Delta \Delta G_{L_{i}\to L_{1}}^{\mathrm{bind}}-\Delta G_{L_{1}}^{\mathrm{bind}}$ . If there is no direct connection between *L*_1_ and some *L*_*i*_, the free energy can be calculated relative to another ligand *L*_*j*_, for which $\Delta G_{L_{j}}^{\mathrm{bind}}$ could be derived directly, according to $\Delta G_{L_{i}}^{\mathrm{bind}}=\Delta \Delta G_{L_{i}\to L_{j}}^{\mathrm{bind}}-\Delta G_{L_{j}}^{\mathrm{bind}}$ . In such cases, we always picked the shortest path available. If there were several equivalent, shortest paths, we calculated the average value along all of them. Following these steps, we re-expressed our results as Δ*G* ^*bind*^ for all ligands of the five model applications.

Two computational studies (Hu et al., 2016; Gapsys et al., 2020) to which we compare to did not report Δ*G* ^*bind*^ values. In these cases, we utilized their tabulated RBFE differences to obtain absolute binding free energy differences as just outlined. For the JNK1, FXa and GAL3 systems we computed RBFE differences for exactly the ligand pairs described in the literature; therefore, we could re-compute any missing Δ*G* ^*bind*^ values exactly as we did for our own results. The absolute binding free energy differences computed in this manner, together with the path used to obtain them, are listed in Supplementary Tables S1–S3. For CDK2 and TYK2, on the other hand, we used a single CC, so our results cannot be directly compared to those reported by Gapsys et al. (2020). In this case, we processed the RBFE differences reported by Gapsys et al. (2020) using exactly the same procedure outlined above for our own results: we searched for the shortest path connecting the reference ligand (and its experimental Δ*G* ^*bind*^) to all other ligands (see Supplementary Tables S4, S5 for full details). For the results of transformato we do not need to search for shortest pathways since all RBFE differences are calculated with respect to the same ligand (we were using a single CC) which was chosen as the reference ligand *L*_1_ (see Supplementary Tables S6, S7).

## 3 Results and discussion

### 3.1 Overview of results for all systems

In total, we computed 76 RBFE differences ${\Delta \Delta G^{bind}}_{L_{i}\to L_{j}}$ , which we also re-expressed as ${\Delta G^{bind}}_{L_{i}}$ . All values, together with the corresponding free energy differences from the earlier studies we compare to (Wang et al., 2015; Hu et al., 2016; Song et al., 2019; Gapsys et al., 2020; He et al., 2020), can be found machine-readable form in the files summary_ddG.csv and summary_dG.csv provided in the SI. The statistical analyses of these data can be found in the Jupyter notebook RBFE_transformato_workbook. ipynb available as SI. A summary of the results is shown in Table 2.

**TABLE 2:** Comparison of a) relative and b) absolute binding free energy differences calculated with transformato, pmx/CGenFF, FEP+ and AMBER-TI with experiment. The root mean squared error (RMSE), the mean absolute error (MAE), both in kcal/mol, as well as the Pearson’s correlation coefficient *R*, and the Spearman’s rank correlation coefficient *ρ* are listed. For the ΔΔ*G* ^*bind*^ results obtained with transformato in a) we also report bootstrapped error estimates for RMSE, MAE, R and *ρ*.

a) **ΔΔG** ^**bind**^										
Transformato					pmx/CGenFF ^*a*^		FEP + ^*b*^		AMBER-TI	
system	RMSE	MAE	*R*	*ρ*	RMSE	MAE	RMSE	MAE	RMSE	MAE
overall	1.18	0.87	0.57	0.48						
	[0.98; 1.38]	[0.72; 1.02]	[0.36; 0.71]	[0.29; 0.64]						
JNK1	0.91	0.68	0.34	0.32	0.95	0.68	1.02	0.78	1.45 ^*c*^	1.15 ^*c*^
	[0.64; 1.17]	[0.51; 0.86]	[-0.02; 0.66]	[-0.02; 0.63]						
FXa	1.23	1.01	0.83	0.71					1.11 ^*d*^	0.72 ^*d*^
	[0.77; 1.69]	[0.70; 1.41]	[0.40; 0.95]	[0.22; 0.99]						
GAL3	0.58	0.50	0.76	0.57	0.61	0.54				
	[0.39; 0.73]	[0.32; 0.68]	[0.08; 0.94]	[-0.17; 0.96]						
CDK2	1.12	0.80	0.63	0.59	1.13	0.84	1.16	0.95	1.16 ^*e*^	0.94 ^*e*^
	[0.69; 1.46]	[0.46; 1.17]	[0.28; 0.88]	[0.08; 0.92]						
TYK2	1.74	1.37	0.42	0.21	1.61	1.33	0.95	0.74	1.29 ^*c*^	1.07 ^*c*^
	[1.26; 2.15]	[0.95; 1.83]	[-0.16; 0.72]	[-0.02; 0.63]						

b) **ΔG** ^**bind**^												
system	Transformato			pmx/CGenFF ^*a*^			FEP + ^*b*^			AMBER-TI		
ΔG	RMSE	MAE	*R*	RMSE	MAE	*R*	RMSE	MAE	*R*	RMSE	MAE	*R*
overall	1.17	0.85	0.73									
JNK1	0.81	0.57	0.60	0.81	0.57	0.66	1.14	1.06	0.85	1.45 ^*c*^	1.07 ^*c*^	0.22 ^*c*^
FXa	1.04	0.92	0.76							0.96 ^*d*^	0.66 ^*d*^	0.83 ^*d*^
GAL3	0.90	0.69	0.59	0.50	0.43	0.90						
CDK2	1.12	0.80	0.61	1.14	0.89	0.41	0.95	0.82	0.52	0.84 ^*e*^	0.72 ^*e*^	0.74 ^*e*^
TYK2	1.74	1.37	0.42	1.87	1.61	0.53	0.58	0.46	0.88	1.27 ^*c*^	1.07 ^*c*^	0.49 ^*c*^

^*a*^ Gapsys et al. (2020), ^*b*^ Wang et al. (2015), ^*c*^ Song et al. (2019), ^*d*^ Hu et al. (2016), ^*e*^ He et al. (2020).

As listed in Table 2, the RMSE (ΔΔ*G* ^*bind*^) of all 76 $\Delta \Delta G_{L_{1}\to L_{2}}^{bind}$ results compared to the experimental binding affinities reported by Wang et al. (2015) is 1.18 kcal/mol and the MAE (ΔΔ*G* ^*bind*^) 0.87 kcal/mol. The Pearsons’s correlation coefficient *R* using all data is 0.57, while Spearman’s *ρ* is 0.48. Seven out of the total $\Delta \Delta G_{L_{1}\to L_{2}}^{bind}$ had an unsigned error (UE) greater than 2 kcal/mol (9.2%). This number is in line with expectation, assuming an underlying Gaussian distribution of the error with *RMSE* (ΔΔ*G* ^*bind*^) = 1.18. For five mutations, the UE was between 1.5 and 2.0 kcal/mol (6.6%), and in 15.8% of the cases the UE was between 1.0 and 1.5 kcal/mol. Thus, 68.4% of the computed RBFE differences were within ±1 kcal/mol of their corresponding experimental value. Plots depicting the correlation between computed and experimental $\Delta \Delta G_{L_{1}\to L_{2}}^{bind}$ results for each of the five model systems are shown in Supplementary Figure S6. Whenever $\Delta \Delta G_{L_{1}\to L_{2}}^{bind}$ results for the same alchemical transformations were reported in previous computational work, the respective data are also included Supplementary Figure S6.

For the absolute binding free energies Δ*G* ^*bind*^ (see Table 2), the performance indicators are very similar to those obtained for ΔΔ*G* ^*bind*^. The RMSE and MAE are almost identical, with the correlation between the results computed by transformato and experiment slightly better (*R* = 0.73). Supplementary Figure S7; Supplementary Table S8 in the SI provide an alternative viewpoint on the quality of the overall results. Supplementary Figure S7 shows a histogram of the deviations between computed and experimental results ${\Delta G^{bind}}_{i,\exp}-{\Delta G^{bind}}_{i}$ . In Supplementary Table S8 the percentage of cases falling within selected ranges of maximal absolute deviation is listed and compared to the expected values drawn from a Gaussian distribution centered about zero (*μ* = 0, i.e., assuming the absence of systematic deviations) with a standard deviation *σ* = RMSE (Δ*G* ^*bind*^) = 1.17 kcal/mol. The plot of this Gaussian is superposed on the histogram (orange, dashed line) in Supplementary Figure S7. Overall, the bell curve fits the data reasonably well. However, one can clearly discern that all results in poor agreement with experiment (results with an UE > 2.5 kcal/mol) are too negative (Supplementary Figure S7) and that their occurrence is slightly higher than expected from a strict Gaussian distribution of errors (Supplementary Table S8). This observation is reflected by the second Gaussian function (solid orange line) plotted in Supplementary Figure S7. This curve was obtained from a fit of the histogram data to a Gaussian; while *σ* remains mostly unchanged, the value of *μ* obtained from the fit is ≈−0.4 kcal/mol. This suggests a small systematic deviation of the computed free energy differences.

As one can see in Supplementary Figure S7 (black, hatched histogram), a very similar trend can be discerned in the pmx/CGenFF results by Gapsys et al. (2020). Since in both studies the same force field was used, this may be indicative of errors resulting from the parameterization. Further, as one can see from the performance indicators (RMSE, MAE, *R*) in Table 2 for each of the five systems, our results for TYK2 are much poorer than for the other four. Indeed, most of the results in poor agreement with experiment were obtained for TYK2 (see below). This is also the case for the pmx/CGenFF results (Gapsys et al., 2020).

### 3.2 Detailed Δ*G* ^*bind*^ results for each system

**FIGURE 3:**
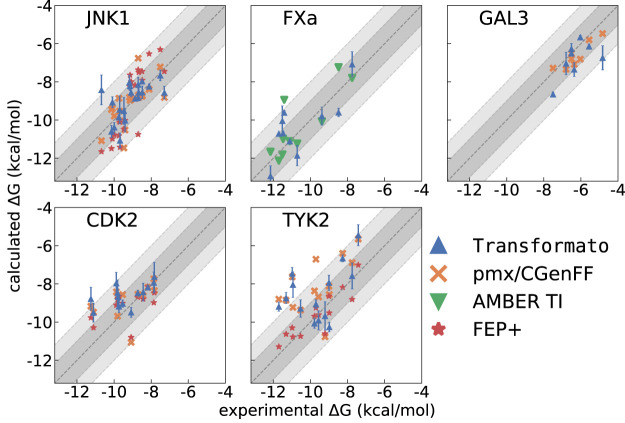
Δ*G* ^*bind*^ calculated with transformato (blue triangles) compared to results obtained by pmx/CGenFF (for JNK1, GAL3, CDK2, TYK2) marked as orange crosses, by FEP+ (for JNK1, CDK2, TYK2) marked as red stars, and by AMBER (FXa) marked as green triangles. The respective RMSE and MAE values are listed in Table 2.

The agreement between computed [this work, pmx/CGenFF (Gapsys et al., 2020), FEP+ (Wang et al., 2015), and AMBER-TI (Hu et al., 2016; Song et al., 2019; He et al., 2020)] and experimentally determined binding affinities is plotted in Figure 3. The graphs complement the statistical descriptors in Table 2.

#### 3.2.1 JNK1

JNK1 is the first of three systems for which we closely followed the transformation paths described in the literature (Wang et al., 2015) to compute RBFE differences $\Delta \Delta G_{L_{1}\to L_{2}}^{bind}$ . In terms of RMSE and MAE (see Table 2) the best results were obtained with transformato and pmx/CGenFF. On the other hand, the highest correlation was obtained with FEP+. Looking at Figure 3, one easily discerns one transformato result which is in very poor agreement with experiment. This is ligand 18652, and the underlying $\Delta \Delta G_{L_{1}\to L_{2}}^{bind}$ calculation causing the error is the transformation of 18631 → 18652 (cf. Supplementary Figure S1). This is a relatively complicated mutation in which several large functional groups need to be turned off to reach the CC state. Specifically, this is an alchemical transformation which, in general, we would try to avoid, but which we carried out to follow the mutation paths used by Wang et al. (2015). Analyzing the raw data, we noted that the phase space overlaps between several intermediate states were poor when using the standard protocol. We, therefore, repeated the full calculation with 10 instead of 5 ns of sampling per state. The longer simulation protocol improved the result dramatically; the $\Delta \Delta G_{L_{1}\to L_{2}}^{bind}$ for the transformation of 18631 → 18652 changed from +1.6 ± 0.48 to −0.11 ± 0.32 kcal/mol, lowering the deviation from experiment from −2.8 to −1.1 kcal/mol. If we include this improved result, our correlation metrics increase (e.g., for Pearson’s *R* from 0.34 to 0.5 for $\Delta \Delta G_{L_{1}\to L_{2}}^{bind}$ and from 0.6 to 0.74 for Δ*G* ^*bind*^). However, our focus was on the quality of the results that can be obtained with transformato using a computationally affordable protocol. Furthermore, no other alchemical transformation had a similarly poor overlap, so we did not recompute any other $\Delta \Delta G_{L_{1}\to L_{2}}^{bind}$ values with the longer simulation protocol.

#### 3.2.2 FXa

FXa is one of two systems not studied by Wang et al. (2015). We computed the $\Delta \Delta G_{L_{1}\to L_{2}}^{bind}$ values reported by Homeyer and Gohlke (2013), but used the protonation states suggested by Hu et al. (2016); hence, we only compare to the latter results. AMBER has a slightly lower RMSE (Δ*G* ^*bind*^) of 0.96 kcal/mol and a MAE (Δ*G* ^*bind*^) of 0.66 kcal/mol, compared to our values of 1.04 and 0.92 kcal/mol. Pearson’s *R* is also better, 0.83 (AMBER) vs. 0.76 (this work). Overall, however, the results of transformato for this system are quite satisfactory. The FXa system was also used for experiments with different charge assignments; these results are not included in Table 2 or Figure 3; see Section 3.4.1 below.

#### 3.2.3 GAL3

The GAL3 system was first studied by Manzoni and Ryde (2018); we followed the mutation paths described there, but compare our results to those obtained with pmx/CGenFF (Gapsys et al., 2020). Our RMSE (Δ*G* ^*bind*^) = 0.90 kcal/mol and MAE (Δ*G* ^*bind*^) = 0.69 kcal/mol are quite good, but higher than those for pmx/CGenFF. Further, Pearson’s *R* for pmx/CGenFF is noticeably higher. However, for the $\Delta \Delta G_{L_{1}\to L_{2}}^{bind}$ results, the performance of transformato and pmx/CGenFF is more similar (see Table 2). This is also the case for Pearson’s *R*, which for pmx/CGenFF is 0.82 (see RBFE_transformato_workbook.ipynb in the SI) and 0.76 for transformato (Table 2).

#### 3.2.4 CDK2

As described in Methods, for CDK2 and TYK2 we employed a single CC; i.e., we followed different mutation paths than those used in the studies we compare to. For CDK2 our CC was based on the smallest ligand of the set (1h1q, see Supplementary Figure S2). Using transformato in this manner takes advantage of the CC/SAI approach; in total only 14 alchemical mutations were required for the 14 ligands studied; this includes the minor changes required for 1h1q → CC. For CDK2, the RMSE (Δ*G* ^*bind*^) and MAE (Δ*G* ^*bind*^) values were reported by all methods (Table 2). The RMSE values lie within a range of 0.3 kcal/mol across the different methods, with the spread of the MAE (0.15 kcal/mol) being even narrower. All methods also have an acceptable Pearson’s *R*. The similarity in performance across all methods can also be seen in Figure 3.

#### 3.2.5 TYK2

As for CDK2, we used a single CC (ejm_31, Supplementary Figure S3), again in contrast to the previously reported approaches, in which more than one mutation path was considered for most of the ligands. As one sees in Table 2 most programs perform relatively poorly for TYK2, the single exception being FEP+ with an RMSE (Δ*G* ^*bind*^) = 0.58 and MAE (Δ*G* ^*bind*^) = 0.46 kcal/mol. Transformato, pmx/CGenFF and AMBER-TI perform significantly worse; the highest deviations were obtained with transformato and pmx/CGenFF. The RMSE (Δ*G* ^*bind*^) for transformato is 1.74 kcal/mol and 1.87 kcal/mol for pmx/CGenFF, and the MAE (Δ*G* ^*bind*^) is also high: 1.37 kcal/mol for transformato and 1.61 kcal/mol for pmx/CGenFF. AMBER-TI performs better with an RMSE (Δ*G* ^*bind*^) of 1.27 kcal/mol and an MAE (Δ*G* ^*bind*^) of 1.07 kcal/mol. This is reflected in Figure 3, where one sees that for transformato, four Δ*G* ^*bind*^ values lie outside the ±2 kcal/mol threshold; this is also the case for pmx/CGenFF. By contrast, for FEP+, only one value lies outside the ±1 kcal/mol threshold. As summarized in Section 3.1 above, only seven $\Delta \Delta G_{L_{1}\to L_{2}}^{bind}$ results deviated from the experimental value by more than 2 kcal/mol; four of these were obtained for TYK2. Clearly, for this system, our results are inferior to those obtained with FEP+ (Wang et al., 2015). However, since the performance of pmx/CGenFF for TYK2 is similarly poor (Gapsys et al., 2020), the CGenFF force field used in both cases might be a contributing factor. We return to the potential role of parameterization in Section 3.4.2.

### 3.3 Additional validation

#### 3.3.1 Use of alternative common cores

**FIGURE 4:**
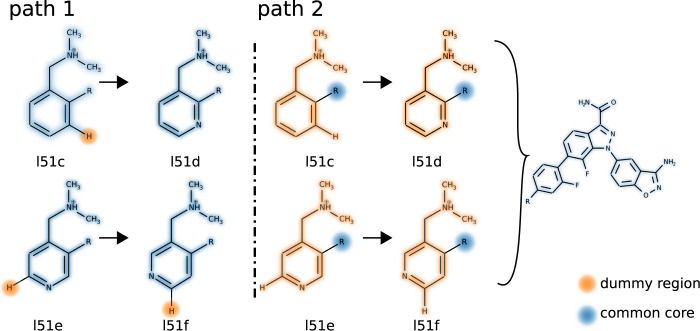
The two pathways for calculating ΔΔ*G* ^*bind*^ (l51c → l51d) and ΔΔ*G* ^*bind*^ (l51e → l51f) of the FXa dataset. Path 1: only a single hydrogen is mutated into a dummy atom (l51c, and l51e, l51f); then, as part of making the CC equivalent, the carbon it is bound to becomes a nitrogen atom (l51d and l51f). Path 2: the dummy region encompasses the compete aromatic ring plus the dimethylammoniomethyl group.

The results presented so far demonstrate the utility of transformato to set up large-scale free energy simulations. To validate the CC/SAI approach further, we calculated two RBFE differences for the FXa model system (l51c → l51d, l51e → l51f) with very different choices for the CCs. The details are shown in Figure 4. As one sees, the mutation consists of either transforming a phenyl ring into a pyridyl group (l51c → l51d), or changing the nitrogen position in a pyridyl group relative to the other substituents (l51e → l51f). Along path 1 (Figure 4, left), we include the aromatic ring in the CC. Thus, only a single hydrogen needs to be transformed into a dummy atom. However, the 2 CCs reached from the two initial states are quite different. Thus, the final stage (stage V, cf. Section 2.4 and Figure 2), transforming the CC reached from one initial state into the other, is involved: the atom types of C and N need to be swapped, which entails the changes of the force field parameters of all associated bonded terms.

The alternative (path 2, Figure 4, center) consists in transforming the full phenyl-/pyridyl ring to dummy atoms. In this case, the dimethylammoniomethyl group present in all four ligands needs to be switched off as well, since one cannot have two disjunct CCs attached to a non-interacting dummy region. Path 2 would be transformato′*s* default mode. However, for the summary reported in Table 2, we used the results obtained along path 1.

The comparison of the detailed results obtained with the two transformations is shown in Table 3. The agreement of $\Delta \Delta G_{L_{1}\to L_{2}}^{bind}$ along the two paths is quite good, although the statistical error along path 2 is significantly larger. This is to be expected, as for each transformation to the CC an aromatic ring plus the dimethylammoniomethyl moiety needed to be mutated to dummy atoms. In practical work with transformato, one would avoid mutations such as those needed for path 2; nevertheless, it is satisfying to see that acceptable results can be obtained even along such a non-optimal path.

**TABLE 3:** $\Delta \Delta G_{L_{1}\to L_{2}}^{\mathrm{bind}}$ for the mutations l51c → l51d, l51e → l51f in the FXa model system along two different paths (see also Figure 4).

Mutation	$\Delta \Delta G_{L_{1}\to L_{2}}^{\mathrm{bind}}$ (path 1)	$\Delta \Delta G_{L_{1}\to L_{2}}^{\mathrm{bind}}$ (path 2)	$\Delta \Delta G_{L_{1}\to L_{2}}^{\mathrm{bind}}$ (exp)
l51c → l51d	4.03 ± 0.7	4.21 ± 1.1	3.36
l51e → l51f	−0.87 ± 0.5	−0.79 ± 1.5	−2.32

#### 3.3.2 Re-computation of selected results with CHARMM/OpenMM and without HMR

All results reported so far were obtained with OpenMM as the underlying MD engine and employing HMR. Recently, Zhang H. et al. (2021) carried out free energy simulations with different time-steps, and with and without HMR. Using CHARMM-GUI’s Free Energy Calculator and AMBER-TI as the free energy tool, they observed good agreement between results obtained with all protocols. We nevertheless wanted to validate these findings for the CC/SAI approach, with a special focus on the correct usage of constraints.

We chose nine ligand pairs from the JNK1, FXa, GAL3 and CDK2 model systems and recomputed the respective $\Delta \Delta G_{L_{1}\to L_{2}}^{bind}$ values *without* HMR and with a “safe” time-step of 1 fs In addition, we also computed these free energy differences using CHARMM/OpenMM as the MD engine. Two of the ligand pairs contain halogens, for which virtual sites (“lone-pairs”) are employed in the CGenFF force field (Gutiérrez et al., 2016). While this poses no difficulty for OpenMM, the use of lone-pairs (regardless of whether they are part of the mutation itself!) seems not supported by CHARMM/OpenMM, so these two $\Delta \Delta G_{L_{1}\to L_{2}}^{bind}$ values could only be computed with OpenMM.

**FIGURE 5:**
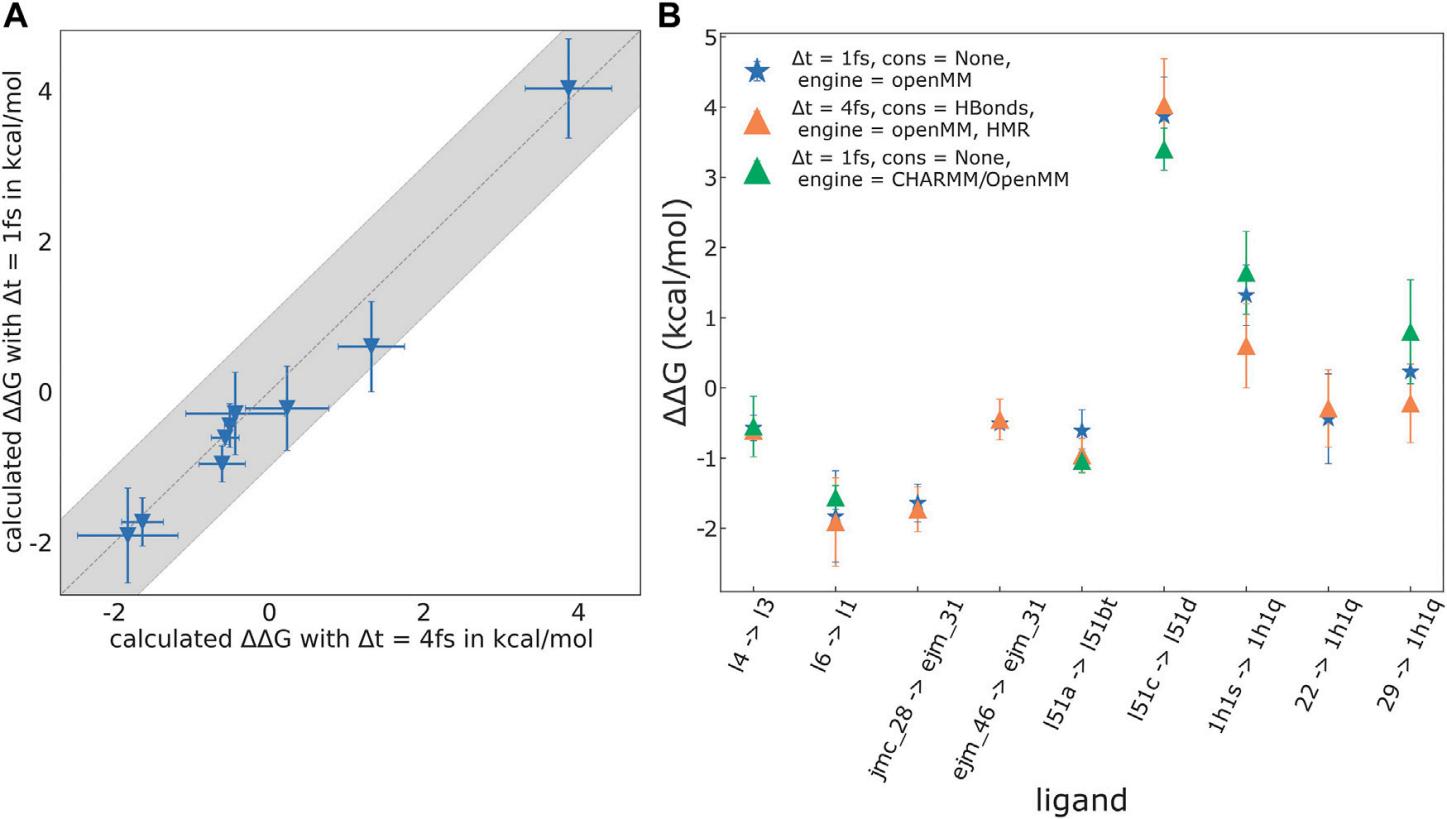
Results for selected mutations recomputed *without* HMR, using OpenMM, as well as CHARMM/OpenMM as the underlying MD engine. The reduced set consists of: GAL3, l4 → l3, l6 → l1; TYK2, jmc_28 → ejm_31, ejm_46 → ejm_31; FXa, l51a → l51d, l51c → l51bt; CDK2, 1h1s → 1h1q, 22 → 1h1q, 29 → 1h1q. **(A)** Plot of results calculated without HMR (timestep Δ*t* = 1 fs, no constraints on the ligand and protein) against results with HMR (Δ*t* = 4 fs, constraints on all bonds involving hydrogen atoms). For the selected mutations RMSE (ΔΔ*G* ^*bind*^) = 0.30 kcal/mol, MAE (ΔΔ*G* ^*bind*^) = 0.21 kcal/mol, Pearson’s *R* = 0.99, and Spearman’s *ρ* = 1.0. **(B)** Plot of the $\Delta \Delta G_{L_{1}\to L_{2}}^{bind}$ results for the mutations of the subset obtained with OpenMM with and without HMR, as well as CHARMM/OpenMM (no HMR). For the results without HMR, using OpenMM and CHARMM/OpenMM as the backend, respectively: RMSE (ΔΔ*G* ^*bind*^) = 0.39 kcal/mol, MAE (ΔΔ*G* ^*bind*^) = 0.35 kcal/mol, Pearson’s *R* = 0.98, and Spearman’s *ρ* = 1.0.

Two plots depicting the results and a detailed list of the mutations studied are shown in Figure 5. We find excellent agreement between the results obtained using OpenMM with and without HMR, with an RMSE/MAE of 0.32/0.23 kcal/mol, respectively. The same is the case for the results obtained with CHARMM/OpenMM compared to the OpenMM results without HMR; here, RMSE (ΔΔ*G* ^*bind*^) = 0.39 and $\text{MAE}\left(\Delta \Delta G_{L_{1}\to L_{2}}^{bind}\right)=0.35$ kcal/mol. Thus, HMR is an excellent tool to speed up free energy simulations, with our settings increasing the throughput by a factor of four (time-step Δ*t* = 4 fs rather than 1 fs; cf. Methods).

When using HMR with a 4 fs time-step, constraints on all bonds containing hydrogen atoms are required to ensure stable simulations. If the equilibrium bond length of a constrained bond is changed during an alchemical mutation, the work exerted by the constraint must be properly accounted for (Boresch and Karplus, 1998). Zhang H. et al. (2021) correctly warn about this difficulty. Presently, transformato avoids this pitfall as follows: First, in the CC/SAI approach one rarely needs to change bond lengths. Second, when such an alchemical mutation is needed, as for the bonds to the junction atom X (cf. Methods), the special atom types used for dummy atoms, as well as for X, are not recognized by OpenMM as hydrogen atoms. For the CHARMM backend, the same can be achieved through atom selections when setting up constraints. Thus, for the currently supported use cases, transformato handles constraints and changes of bond lengths arising in alchemical mutations correctly.

### 3.4 Influences of force field and charge assignment

#### 3.4.1 Charge assignment and tautomerism

One of the ligands in the FXa dataset can exist in two tautomeric forms, labelled l51b and l51bt by Hu et al. (2016). The computed $\Delta \Delta G_{L_{1}\to L_{2}}^{bind}$ for the transformation l51a → l51b deviated by nearly 3 kcal/mol from the experimental result, whereas the computational result for l51a → l51bt agreed well with experiment (Hu et al., 2016); see also Table 4. By contrast, our calculations give very similar free energy differences for both tautomeric states, −1.23 kcal/mol and −1.07 kcal/mol, respectively, both in good agreement with the experimental value of −1.45 kcal/mol (see Table 4).

**TABLE 4:** $\Delta \Delta G_{L_{1}\to L_{2}}^{\mathrm{bind}}$ in kcal/mol for the alchemical transformations of l51a into the two tautomeric forms of ligand l51b/l51bt of the FXa dataset. TF denotes results obtained with transformato. Values for $\Delta \Delta G_{L_{1}\to L_{2}}^{\text{AMBER}}$ and $\Delta \Delta G_{L_{1}\to L_{2}}^{\text{exp}}$ were taken from Hu et al. (2016).

Mutation	$\Delta \Delta G_{L_{1}\to L_{2}}^{TF}$	$\Delta \Delta G_{L_{1}\to L_{2}}^{\mathrm{AMBER}}$	$\Delta \Delta G_{L_{1}\to L_{2}}^{\exp}$
l51a → l51b (CGenFF charges)	−1.23 ± 0.3	1.44 ± 0.2	−1.45
l51a → l51b (AMBER charges)	0.76 ± 0.3		
l51a → l51bt (CGenFF charges)	−1.07 ± 0.3	−1.19 ± 0.1	
l51a → l51bt (AMBER charges)	−0.56 ± 0.1		

Since on the one hand tautomers and ionization states are a source of error in free energy simulations (Cournia et al., 2021) and the disagreement between the two calculational results is surprisingly large, we decided to investigate this further. It seems reasonable to focus on electrostatic interactions. Therefore, we made the partial charges of the ligand more similar to the parameterization used by Hu et al. (2016), i.e., the AMBER force field family, atomic charges prepared with the AM1-BCC approach (Jakalian et al., 2000). We replaced the charges from ParamChem/CGenFF with charges from the ACPYPE server (Silva and Vranken, 2012). When repeating the RBFE calculations for the two transformations (l51a → l51 b/t) with these chimeric parameters (CGenFF force field, but AM1-BCC charges), the results were much closer to the values reported by Hu et al. (2016), with a positive sign for the mutation l51a → l51b and a negative one for the favored mutation l51a → l51bt (see Table 4).

While mixing parameters as described above is *not* recommended, the results make clear the origin of the rather different free energy predictions obtained in this particular case by transformato and AMBER (Hu et al., 2016), respectively. The rather dissimilar results for the two charge models highlight the difficulty in relying on additive force fields to predict the preferred tautomeric state.

#### 3.4.2 Observations concerning the influence of the force field

The data in Table 2 and the plots shown in Figure 3 demonstrate the good overall agreement between results obtained by transformato with results of previous studies in the literature. For four out of five model applications, the computed binding free energy differences are quite comparable between different programs and force fields. As already described in Sections 3.1 and 3.2, the single exception are the results for TYK2. Here, transformato performed worse than FEP+ (Wang et al., 2015), and our results showed high deviations compared to experiment. However, TYK2 seems a challenging system for computational methods. The results obtained with AMBER-TI (Song et al., 2019) are already in poorer agreement with experiment, with Pearson’s *R* = 0.49 compared to *R* = 0.88 for FEP+. The RMSE (Δ*G* ^*bind*^) and MAE (Δ*G* ^*bind*^) of pmx/CGenFF are similarly high as the values obtained with transformato (see Table 2). Recently, it was shown that for TYK2 a refinement of alchemical free energy differences by a physics-based machine learning potential improved the agreement with experiment significantly (Rufa et al., 2020). The comparable performance of transformato and of the results by Gapsys et al. (2020) may indicate shortcomings in the CGenFF parameters of the ligand in the case of the TYK2 application, as this force field was used in both studies.

The ParamChem/CGenFF parameterization procedure reports two “penalty scores,” one for the partial charges assigned, and one for the bonded parameters. These scores indicate the amount of analogy that could be established during the parameter assignment. High values do not automatically mean a poor quality of the parameters, but they are a warning to the user that further inspection and optimization may be needed.^1^ Looking at the penalty scores reported for the sets of ligands used in this work, we find, e.g., for CDK2, a system for which transformato’*s* agreement with experiment is very good, maximal charge penalties between 21 and 43, and maximal bonded parameter penalties between 45 and 89. For TYK2, on the other hand, the largest charge penalty is 142. Furthermore, for the ligands containing a cyclopropyl moiety (ejm46, jmc23, jmc27, jmc28, jmc30; see Supplementary Figure S3) the maximal parameter penalties range from 114 to 225. These are exactly the ligands for which we obtained the largest deviations from the experimental binding free energy differences (UE > 2.5 kcal/mol) and that contribute to the systematic offset of ≈−0.4 kcal/mol for the average deviation between results obtained with transformato and experiment (cf. Section 3.1 and Supplementary Figure S7). While the penalty scores for the three other systems, JNK1, FXa, and GAL3, are higher than those for CDK2, they are lower than those for TYK2.

The cyclopropyl-containing ligands of the TYK2 dataset have high parameter penalties, as just mentioned, but acceptable charge penalties, in particular for the atoms of the cyclopropyl moiety itself. This is not surprising, as cyclopropane is part of the training set, so reference partial charges for this group are available. However, the partial charges are exactly the same as those for normal alkyl groups. According to the FreeSolv database (Mobley and Guthrie, 2014), the solvation free energies of propane and cyclopropane are +2.0 and +0.75 kcal/mol, respectively. In previous work (Fleck et al., 2021), we obtained +2.33 kcal/mol for the solvation free energy of propane using the CGenFF parameters, a value in good agreement with experiment. To probe the quality of the cyclopropane parameters, we therefore computed its solvation free energy difference, following the protocol by Fleck et al. (2021), and obtained Δ*G*_*solv*_ = +2.17 ± 0.07 kcal/mol. Thus, the CGenFF parameters make cyclopropane more hydrophobic than it should be. We note in passing that the solvation free energies using AMBER parameters reported in the FreeSolv database are +2.50 and +2.48 kcal/mol for propane and cyclopropane, respectively, i.e., quite comparable to the CHARMM results. While all of the above is “circumstantial evidence,” the high parameter penalties for the cyclopropyl-containing ligands, combined with the rather poor agreement between the computed and experimental solvation free energy of cyclopropane itself, suggest that insufficiencies of the force field may indeed at least partially responsible for the poor performance observed for TYK2.

## 4 Concluding discussion

In the study introducing transformato (Wieder et al., 2022), we demonstrated that one can achieve very high accuracy with the CC/SAI approach for (relative) solvation free energy differences. Our present results demonstrate that the approach can be extended to large-scale RBFE calculations and that, overall, the agreement with related approaches (Wang et al., 2015; Song et al., 2019; Gapsys et al., 2020; He et al., 2020; Petrov, 2021) is good.

Transformato is developed as an open source project (see Code and Data Availability below), and it has reached a state in which it is possible for others to use/test it. Obviously, this is work in progress and several aspects of transformato can and should be improved. For JNK1, we found one mutation, for which phase space overlaps between some neighboring states were low. While in this particular case the best solution would be to avoid this specific mutation path, such situations should be flagged automatically. Some of our results, in particular those for the TYK2 dataset, highlight the sensitivity of the accuracy of RBFE calculations to the force field parameters. Other sources of systematic errors that can arise in free energy simulations of protein–ligand systems include, e.g., differences in binding modes of ligands not captured by the simulations, handling of trapped waters and choosing the correct ionization/tautomerization states; for additional details we refer the reader to Mey et al. (2020) and Cournia et al. (2021) and the references listed therein. One methodological aspect more specific to the CC/SAI approach is the following: Situations may arise in which the CC is significantly smaller than the physical ligand; hence, it may be bound much less tightly and move around in the binding pocket. Complications resulting from this unwanted flexibility can be prevented by adding restraints to keep the CC in a position and orientation with respect to the receptor that resembles the physical protein–ligand complex. We did not detect indications of such problems in the results reported here, but work towards implementing such restraints is currently ongoing.

The CC/SAI approach is well suited for situations in which a large portion of the ligands under consideration is identical and when the structural differences are located in one or more functional groups. Inefficiencies may arise if the CC is significantly smaller than either of the two ligands. In such a case, for both ligands, a large number of atoms needs to be mutated into dummy atoms, which not only increases the computational cost, but also lowers accuracy and precision. Such is the situation encountered for path 2 in Section 3.3.1, as reflected by the noticeably higher statistical uncertainty for the results obtained along this route. The example, however, also highlights that the automatic CC assignment of transformato can be overridden, as was done along path 1. In such cases, stage (V) (cf. Section 2.4) is more involved, but transformato supports the necessary setup.

The CC/SAI approach implemented in transformato completely separates the logistics of setting up the alchemical mutation from the underlying MD simulations. One benefit of this is performance; the plain MD simulations can use the fastest available code paths. On average, using OpenMM, a full calculation of $\Delta \Delta G_{L_{1}\to L_{2}}^{bind}$ took approximately 1 day on a single mid-range, consumer grade GPU (NVIDIA RTX3070). As the required MD simulations are completely independent, they can be distributed between several computers with linear speedup. Further, adding support for additional MD programs should not be difficult. Maybe even more important, the full functionality of the underlying MD engine can be used. E.g., with OpenMM, we can use the CGenFF parameters that place lone-pairs on the halogens (Gutiérrez et al., 2016)^2^. Further extending transformato to the Drude polarizable force field, given the existing support in OpenMM (Huang et al., 2018) and CHARMM-GUI (Kognole et al., 2021), should be straightforward.

Setting up alchemical transformations with transformato relies on CHARMM-GUI (Jo et al., 2008; Lee et al., 2016); in other words, in principle, RBFE differences can be computed for any system that can be handled by CHARMM-GUI, such as membrane proteins, etc. Thus, particularly for academic users, transformato provides a low-cost/low-barrier entry to large-scale RBFE calculations.

## 5 Code and data availability

• Python package used in this work (release v0.2): https://github.com/wiederm/transformato.

## Data Availability

The datasets presented in this study can be found in online repositories. The names of the repository/repositories and accession number(s) can be found in the article/Supplementary Material.

## Footnotes

1 See, e.g., the frequently asked questions at https://cgenff.umaryland.edu/commonFiles/faq.php

2 Supporting lone-pairs required a patch to parmed (Shirts et al., 2017), which is available from https://github.com/JohannesKarwou/ParmEd. It should be available in the next official parmed release.
