# Supplementary material for: Relative binding free energy calculations with transformato: A molecular dynamics engine-independent tool
Source: Front Mol Biosci. 2022 Sep 6;9:954638. doi: 10.3389/fmolb.2022.954638 (PMC9485484; doi:10.3389/fmolb.2022.954638)
Supplement: Supplementary file 1 [file DataSheet1.pdf]

Supplementary Material

This document contains supplementary figures and tables. In addition, a zip-archive is available, containing our raw results in machine-readable form, as well as the Jupyter notebook with which additional statistical analyses were carried out.

Contents of the zip-archive:

- `summary_ddG.csv`
- `summary_dG.csv`
- `RBFE_transformato_workbook.ipynb`

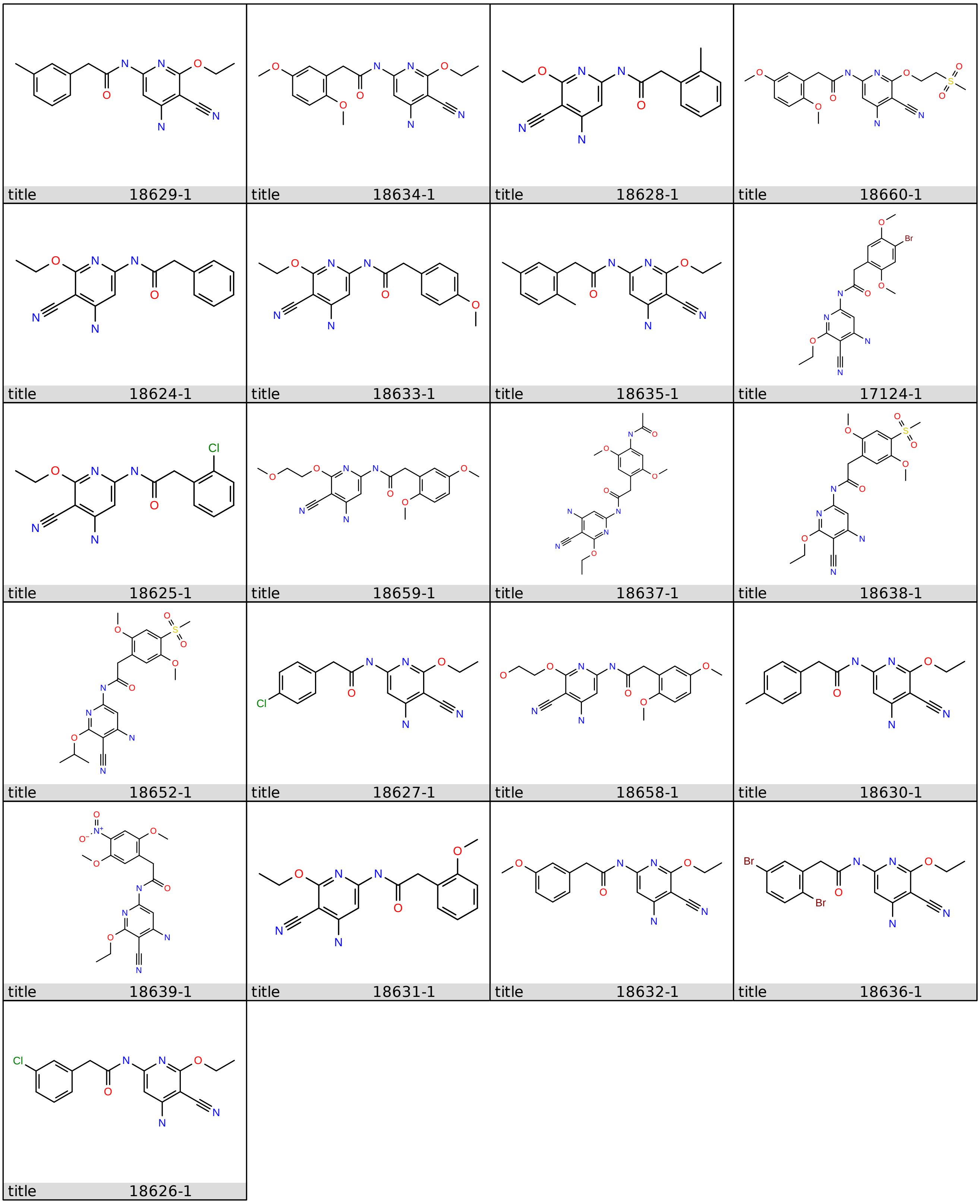

Figure S1. Structures for the ligands investigated for the JNK1 system with their corresponding abbreviations (Wang et al., 2015). Different CCs were used depending on the mutation studied.

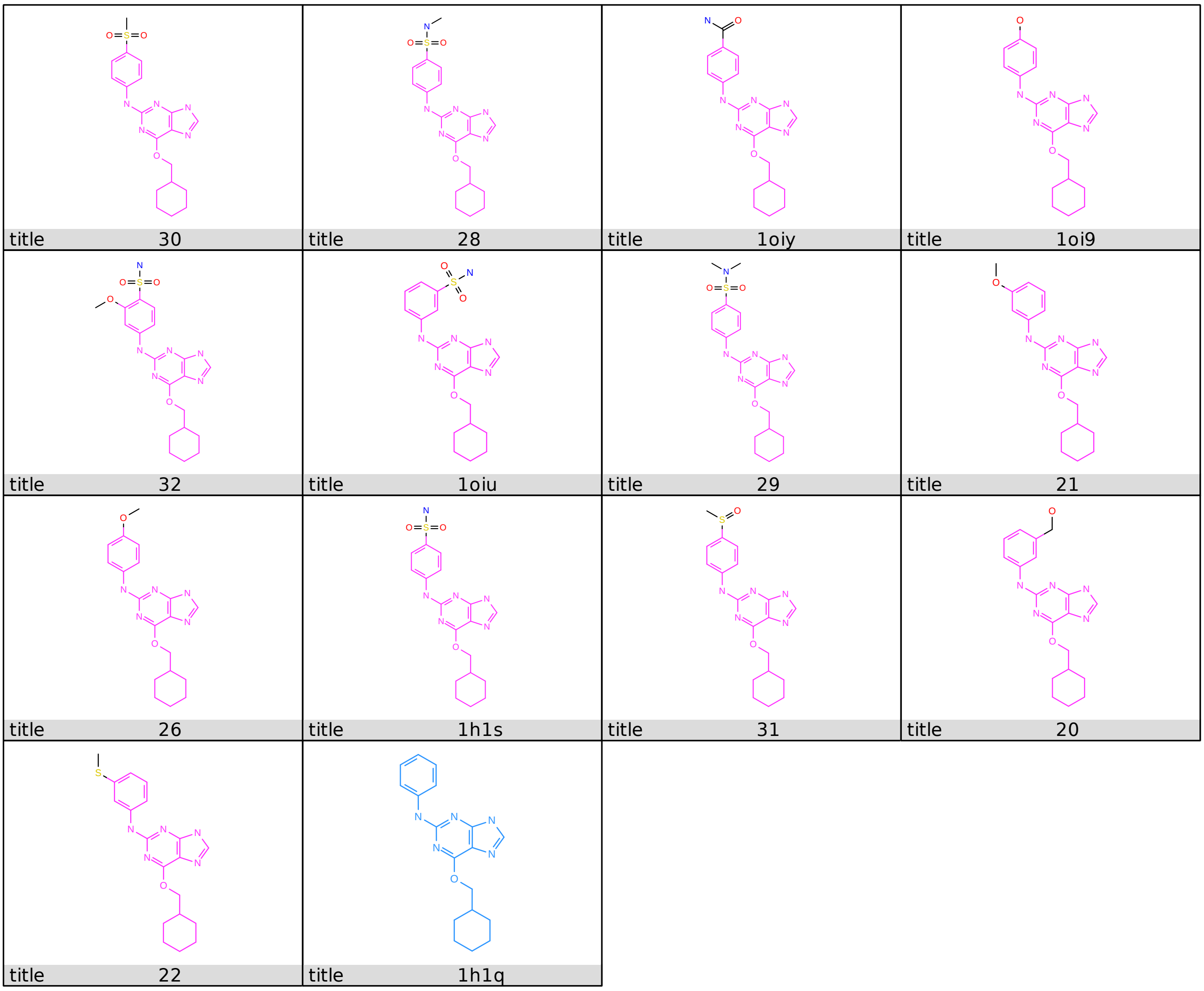

Figure S2. Structures for the ligands investigated for the CDK2 system with their corresponding abbreviations (Wang et al., 2015). Ligand 1h1q, highlighted in blue, is the reference structure for the consensus common core (CC) used in this work.

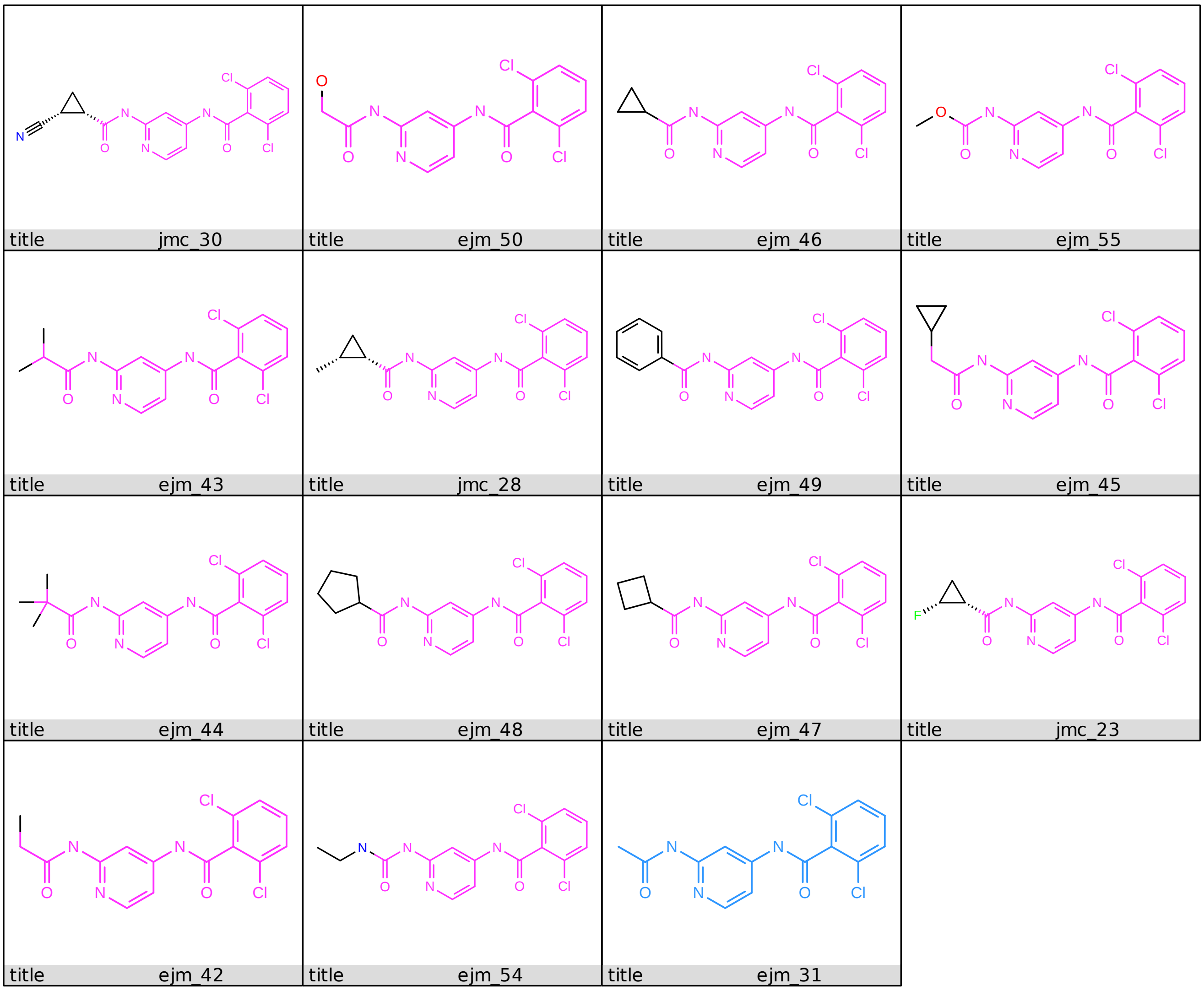

Figure S3. Structures for the ligands investigated for the TYK2 system with their corresponding abbreviations (Wang et al., 2015). Ligand ejm_31, highlighted in blue, is the reference structure for the consensus common core (CC) used in this work.

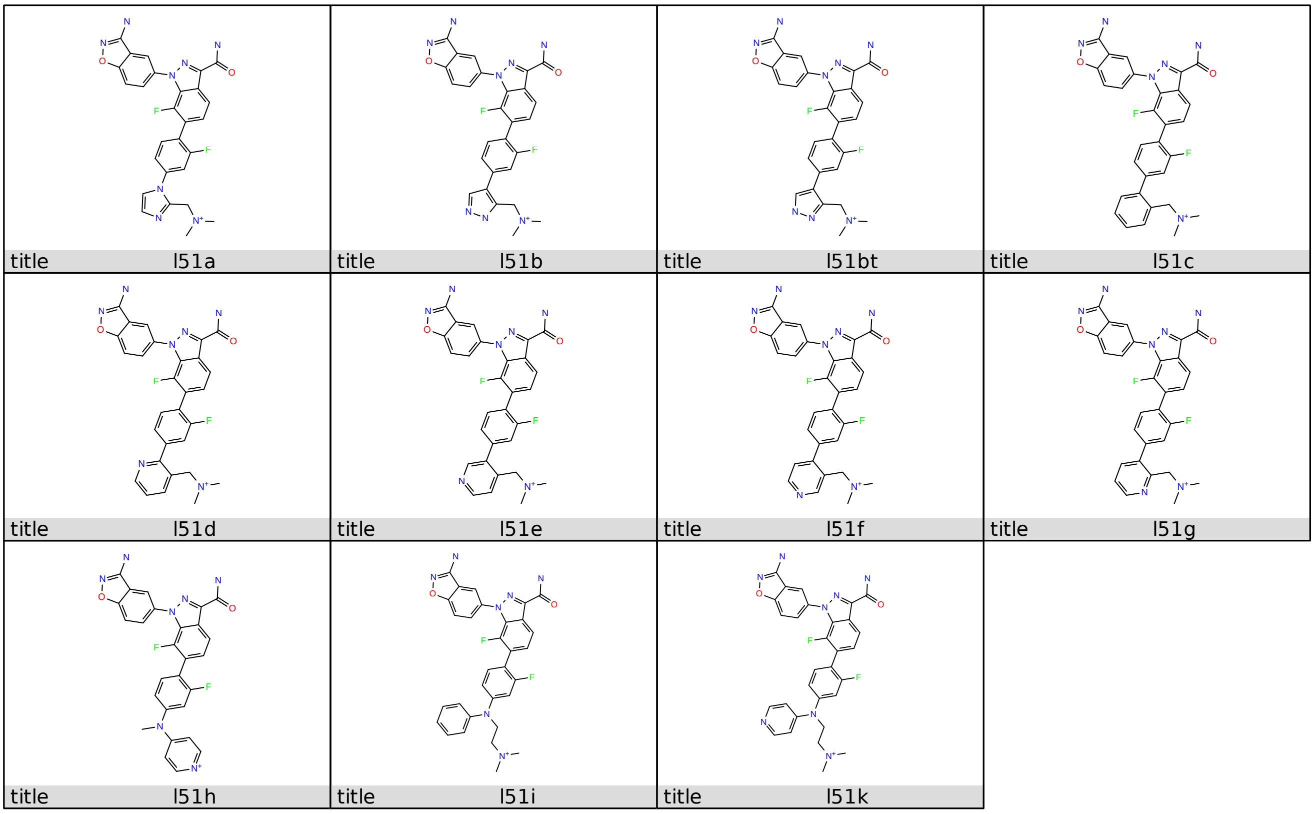

Figure S4. Structures for the ligands investigated for the XFa system with their corresponding abbreviations (Homeyer and Gohlke, 2013). Different CCs were used depending on the mutation studied.

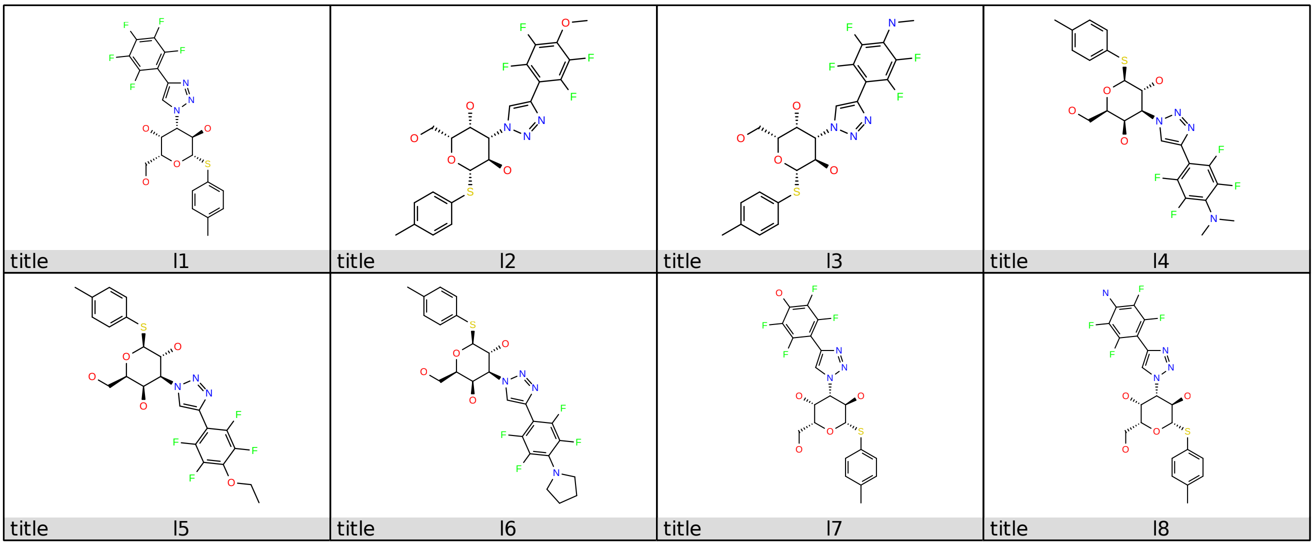

Figure S5. Structures for the ligands investigated for the Galectin–3 system with their corresponding abbreviations (Gapsys et al., 2020). Different CCs were used depending on the mutation studied.

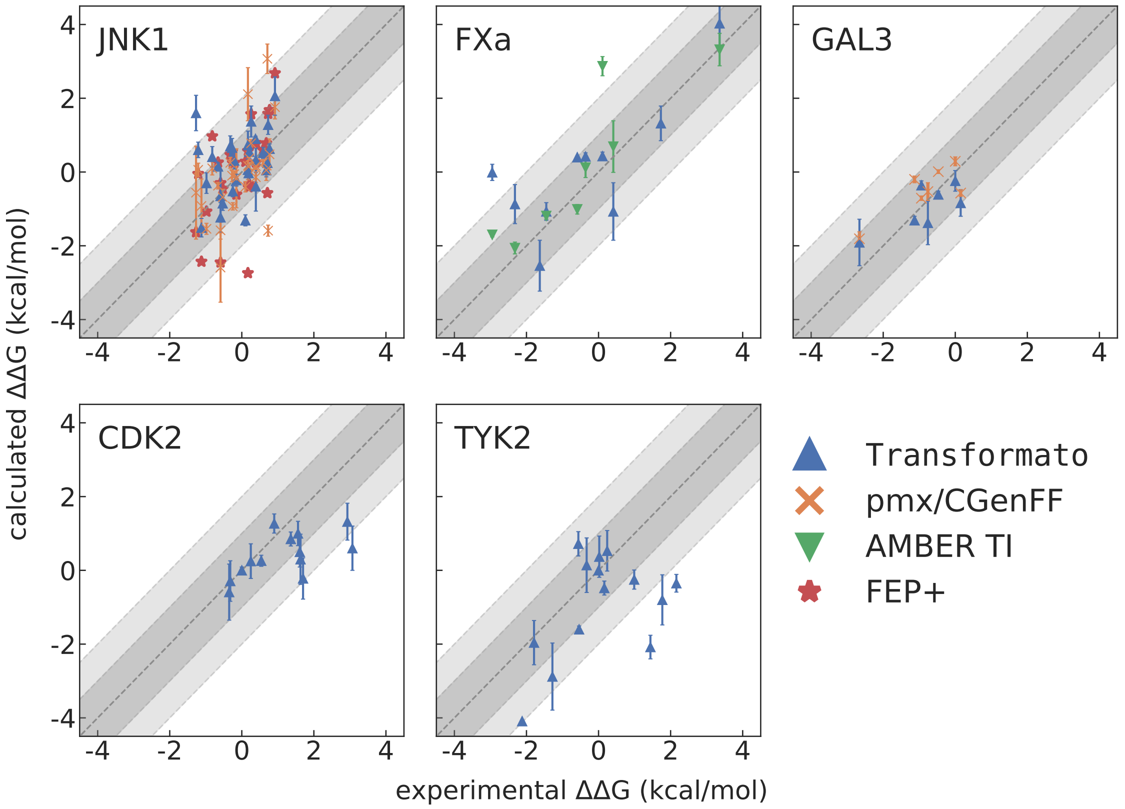

Figure S6. $\Delta\Delta G^{bind}_{L_1 \to L_2}$ obtained with `Transformato` (blue triangles) compared to results obtained by pmx/CGenFF for JNK1, GAL3 (orange crosses), by FEP+ for JNK1 (red stars), and by AMBER TI for XFa (green triangles). The corresponding RMSE and MAE values can be found in Table 2a of the main manuscript.

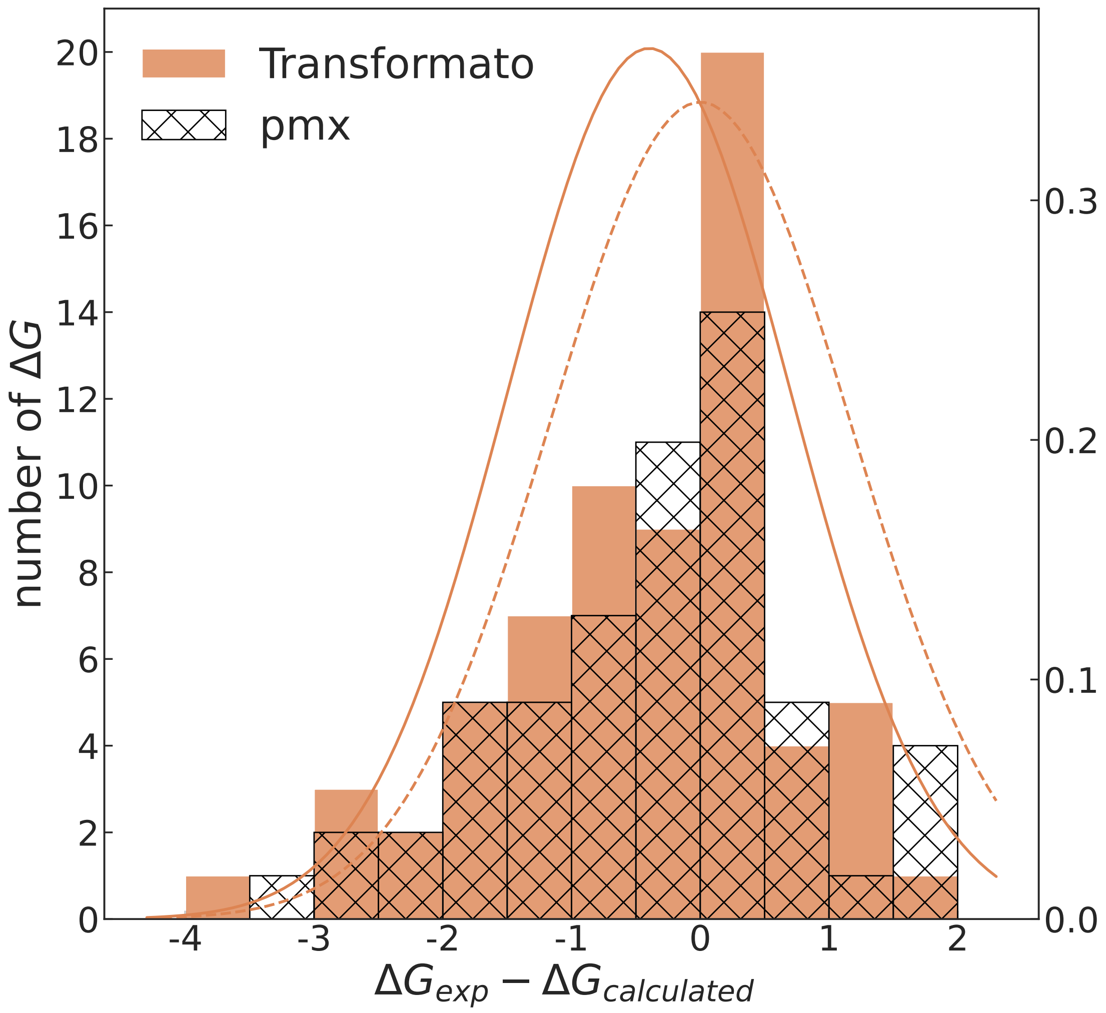

Figure S7. Histogram of deviations between the computed binding free energies ΔG^{bind} obtained by `Transformato` (orange) and pmx/CGenFF (black, hatched histogram) and experiment. The data obtained with `Transformato` are superposed with two Gaussian distributions. Solid orange line: $\mu = 0$, $\sigma = 1.17$ (1.17 kcal/mol is the RMSE of the predicted binding free energies for `Transformato`). Dashed orange line: Best fit Gaussian $\mu = -0.40$, $\sigma = 1.09$. For clarity, the analogous graphs for pmx/CGenFF are not shown; the parameters for the fitted Gaussian is $\mu = -0.41$, $\sigma = 1.15$

Table S1. Comparison of experimental ΔG values with calculated ΔG values for pmx/CGenFF and `Transformato` (TF) for the JNK1 system. The computed $\Delta\Delta G$ values for pmx/CGenFF were taken from Gapsys et al. (2020)

	exp. ΔG^a	calc. ΔG (pmx/CGenFF)	UE (pmx/CGenFF)	calc. ΔG TF	UE TF	Calculation of ΔG
18628	-8.70	-8.70 ± 0.38	0.00	-8.81 ± 0.10	0.11	ΔG(18626)+ΔΔG(18626→18628)
18624	-8.49	-8.76 ± 0.03	0.27	-7.97 ± 0.07	0.52	ΔG(18626)+ΔΔG(18626→18624)
18639	-9.74	-8.86 ± 0.10	0.88	-9.85 ± 0.15	0.11	ΔG(18634)-ΔΔG(18639→18634)
18660	-8.70	-6.76 ± 0.72	1.94	-8.14 ± 0.38	0.56	ΔG(18626)+ΔΔG(18626→18660)
18630	-9.14	-8.97 ± 0.16	0.17	-8.21 ± 0.25	0.93	ΔG(18626)+ΔΔG(18626→18630)
18632	-9.08	-8.93 ± 0.09	0.15	-8.56 ± 0.23	0.52	ΔG(18626)+ΔΔG(18626→18632)
18636	-7.51	-7.22 ± 0.15	0.29	-7.66 ± 0.28	0.15	ΔG(18624)-ΔΔG(18636→18624)
18652	-10.68	-11.08 ± 1.32	0.40	-8.42 ± 0.78	2.26	ΔG(18631)+ΔΔG(18631→18652)
17124	-9.68	-10.04 ± 0.75	0.36	-11.07± 0.38	1.39	ΔG(18634)-ΔΔG(17124→18634)
18635	-7.29	-8.82 ± 0.24	1.53	-8.56 ± 0.33	1.27	ΔG(18624)-ΔΔG(18635→18624)
18627	-8.48	-8.71 ± 0.09	0.23	-8.55 ± 0.33	0.07	ΔG(18626)+ΔΔG(18626→18627)
18637	-10.14	-9.45 ± 0.81	0.70	-10.95± 0.32	0.46	ΔG(18634)+ΔΔG(18634→18637)
18634	-9.99	-9.79 ± 0.73	0.20	-10.38± 0.25	0.39	ΔG(18626)+ΔΔG(18626→18634)
18629	-8.67	-8.82 ± 0.77	0.15	-8.56 ± 0.43	0.11	ΔG(18624)-ΔΔG(18635→18624)
18631	-9.41	-10.52 ± 0.40	1.11	-10.02± 0.61	0.61	ΔG(18624)-ΔΔG(18631→18624)
18633	-9.17	-8.81 ± 0.29	0.36	-8.02 ± 0.38	1.15	ΔG(18624)-ΔΔG(18633→18624)
18658	-9.70	-9.53 ± 0.76	0.17	-9.46 ± 0.73	0.24	ΔG(18638)+ΔΔG(18638→18658)
18638	-10.09	-9.39 ± 0.74	0.70	-9.07 ± 0.29	1.02	ΔG(18634)-ΔΔG(18638→18634)
18625	-8.11	-8.39 ± 0.42	0.28	-8.24 ± 0.13	0.13	ΔG(18626)+ΔΔG(18626→18625)
18659	-9.47	-11.46 ± 0.94	1.99	-9.52 ± 0.71	0.05	ΔG(18626)+ΔΔG(18626→18659)
**18626**	**-8.87**	**-8.87**	**0.00**	**-8.87**	**0.00**	**experimental value**

[a] as reported by Wang et al. (2015)

Table S2. Comparison of experimental ΔG values with calculated ΔG values for pmx/CGenFF and `Transformato` (TF) for the XFa system. The computed $\Delta\Delta G$ values for pmx/CGenFF were taken from Hu et al. (2016). The unsigned error (UE) is given for all values besides the reference structure.

	exp. ΔG^a	ΔG (pmx/CGenFF)	UE (pmx/CGenFF)	ΔG (TF)	UE (TF)	Calculation of ΔG
l51a	-10.71	-11.23 ± 0.57	0.81	-11.52 ± 0.53	0.81	ΔG(l51g)+ΔΔG(l51g→l51a)
l51bt	-12.15	-11.66 ± 0.75	0.27	-12.48 ± 0.54	0.33	ΔG(l51a)+ΔΔG(l51a→l51bt)
**l51c**	**-11.11**	**-11.11**	**0.00**	**-11.11**	**0.00**	**experimental value**
l51d	-7.75	-7.79 ± 0.44	0.04	-7.08 ± 0.66	0.67	ΔG(l51c)+ΔΔG(l51c→l51d)
l51e	-9.38	-10.07 ± 0.20	0.68	-9.79 ± 0.47	0.41	ΔG(l51c)+ΔΔG(l51c→l51e)
l51f	-11.70	-12.12 ± 0.13	0.42	-10.71 ± 0.03	0.99	ΔG(l51c)+ΔΔG(l51c→l51f)
l51g	-11.47	-10.99 ± 0.27	0.48	-10.69 ± 0.10	0.78	ΔG(l51c)+ΔΔG(l51c→l51g)
l51h	-11.51	-11.80 ± 0.70	0.29	-10.04 ± 0.78	1.47	ΔG(l51c)-ΔΔG(l51h→l51c)
l51i	-8.48	-7.23 ± 0.06	1.25	-9.60 ± 0.22	1.12	ΔG(l51k)-ΔΔG(l51i→l51k)
l51k	-11.41	-8.94 ± 0.26	2.48	-9.61 ± 0.11	1.80	ΔG(l51h)+ΔΔG(l51h→l51k)

[a] as reported by Wang et al. (2015)

Table S3. Comparison of experimental ΔG values with calculated ΔG values for pmx/CGenFF and `Transformato` (TF) for the Galectin–3 system. The computed $\Delta\Delta G$ values for pmx/CGenFF were taken from Hu et al. (2016). The unsigned error (UE) is given for all values besides the reference structure.

	exp. ΔG^a	ΔG (pmx/CGenFF)	UE (pmx/CGenFF)	ΔG (TF)	UE (TF)	Calculation of ΔG
l1	-7.50	-7.28 ± 0.13	0.22	-8.65 ± 0.38	1.15	ΔG(l7)+ΔΔG(l7→l1)
**l2**	**-6.51**	**-6.51**	**0.00**	**-6.51**	**0.00**	**experimental value**
l3	-6.51	-6.80 ± 0.12	0.29	-6.27 ± 0.28	0.24	ΔG(l2)-ΔΔG(l3→l2)
l4	-6.03	-6.81 ± 0.13	0.77	-5.66 ± 0.40	0.37	ΔG(l3)-ΔΔG(l4→l3)
l5	-5.56	-5.80 ± 0.06	0.23	-6.14 ± 0.12	0.58	ΔG(l2)-ΔΔG(l5→l2)
l6	-4.83	-5.47 ± 0.23	0.64	-6.74 ± 0.74	1.91	ΔG(l1)-ΔΔG(l6→l1)
l7	-6.36	-7.08 ± 0.09	0.71	-7.35 ± 0.36	0.98	ΔG(l2)+ΔΔG(l2→l7)
l8	-6.80	-7.34 ± 0.27	0.54	-7.04 ± 0.71	0.24	ΔG(l4)+ΔΔG(l4→l8)

[a] taken from Peterson (2018)

Table S4. Comparison of experimental Δ G values with the corresponding calculated ΔG value for the CDK2 system. The formula how ΔG was derived from the ΔΔG values as reported by Gapsys et al. (2020) by pmx/CGenFF is given in the last column. The unsigned error (UE) is given for all values besides the reference structure.

	exp. ΔG^a	ΔG (pmx/CGenFF)	UE (pmx/CGenFF)	Calculation of Δ G
20	-8.72	-8.55 ± 0.07	0.17	ΔG(1h1q)-ΔΔG(20→1h1q)
21	-7.83	-8.35 ± 0.59	0.52	ΔG(17)+ΔΔG(17→21)
22	-7.86	-7.73 ± 0.56	0.13	ΔG(17)+ΔΔG(17→22)
26	-8.43	-8.62 ± 0.22	0.19	ΔG(1h1q)-ΔΔG(26→1h1q)
28	-11.11	-9.48 ± 0.41	1.63	ΔG(26)-ΔΔG(28→26)
29	-9.88	-8.42 ± 0.25	1.46	(ΔG(26)-ΔΔG(29→26))+(ΔG(1oiy)+ΔΔG(1oiy→29))]/2
30	-9.81	-9.69 ± 0.26	0.12	(ΔG(26)-ΔΔG(30→26))+(ΔG(31)+ΔΔG(30→31))]/2
31	-9.54	-8.56 ± 0.52	0.98	ΔG(1oiy)+ΔΔG(1oiy→31)
**1h1q**	**-8.18**	**-8.18**	**0.00**	**experimental value**
1h1s	-11.25	-9.17 ± 0.27	2.08	(ΔG(26)-ΔΔG(1h1s→26))+(ΔG(1oiy)+ΔΔG(1h1s→1oiy))]/2
1oi9	-9.74	-8.57 ± 0.23	1.17	(ΔG(26)+ΔΔG(26→1oi9))+(ΔG(1oiy)+ΔΔG(1oiy→1oi9))]/2
1oiu	-9.08	-11.06± 0.35	1.98	ΔG(1h1q)-ΔΔG(1oiu→1h1q)
1oiy	-9.79	-8.66 ± 0.49	1.13	ΔG(1h1q)-ΔΔG(1oiy→1h1q)

[a] as reported by Wang et al. (2015)

Table S5. Comparison of experimental Δ G values with the corresponding calculated ΔG value for the TYK2 system. The formula how ΔG was derived from the ΔΔG values as reported by Gapsys et al. (2020) by pmx/CGenFF is given in the last column. The unsigned error (UE) is given for all values besides the reference structure.

	exp. ΔG^a	ΔG (pmx/CGenFF)	UE (pmx/CGenFF)	Calculation of Δ G
**ejm_31**	**-9.54**	**-9.54**	**0.00**	**experimental value**
ejm_42	-9.78	-8.37 ± 0.27	1.41	[(ΔG(ejm48)-ΔΔG(ejm42→ejm48))+(ΔG(ejm45)+ΔΔG(ejm45→ejm42))]/2
ejm_43	-8.26	-6.40 ± 0.61	1.86	ΔG(ejm31)+ΔΔG(ejm31→ejm43)
ejm_44	-7.42	-5.65 ± 0.45	1.77	[(ΔG(ejm42)-ΔΔG(ejm44→ejm42))+(ΔG(ejm55)-ΔΔG(ejm44→ejm55))]/2
ejm_45	-9.56	-8.68 ± 0.40	0.88	ΔG(ejm31)+ΔΔG(ejm31→ejm45)
ejm_46	-11.31	-8.85 ± 0.07	2.46	ΔG(ejm31)+ΔΔG(ejm31→ejm46)
ejm_47	-9.70	-6.71 ± 0.22	2.99	ΔG(ejm31)-ΔΔG(ejm47→ejm31)
ejm_48	-9.00	-8.12 ± 0.20	0.88	ΔG(ejm31)+ΔΔG(ejm31→ejm48)
ejm_49	-7.75	-6.89 ± 0.17	0.86	ΔG(ejm31)-ΔΔG(ejm49→ejm31)
ejm_50	-8.98	-8.56 ± 0.31	0.42	ΔG(ejm49)+ΔΔG(ejm50)
ejm_54	-10.53	-9.37 ± 0.33	1.16	[(ΔG(ejm42)+ΔΔG(ejm42→ejm54))+(ΔG(ejm55)-ΔΔG(ejm55-ejm54))]/2
ejm_55	-9.21	-10.77 ± 1.17	1.56	[(ΔG(ejm43)-ΔΔG(ejm43→ejm55))+(ΔG(ejm47)-ΔΔG(ejm47-ejm55))]/2
jmc_23	-11.70	-8.80 ± 0.13	2.90	ΔG(ejm46)-ΔΔG(jmc23→ejm46)
jmc_28	-10.98	-7.63 ± 0.31	3.35	ΔG(ejm31)+ΔΔG(ejm31→jmc28)
jmc_30	-10.94	-9.23 ± 0.56	1.71	ΔG(jmc28)+ΔΔG(jmc28→jmc30)

[a] as reported by Wang et al. (2015)

Table S6. Comparison of experimental Δ G values with the corresponding calculated ΔG value for the CDK2 system. The formula how ΔG was derived from the ΔΔG values as reported by `Transformato` is given in the last column. The unsigned error (UE) is given for all values besides the reference structure.

	exp. ΔG^a	ΔG (TF)	UE (TF)	Calculation of Δ G
20	-8.72	-8.48 ± 0.15	0.24	ΔG(1h1q)-ΔΔG(20→1h1q)
21	-7.83	-7.63 ± 0.76	0.20	ΔG(1h1q)-ΔΔG(21→1h1q)
22	-7.86	-7.93 ± 0.55	0.07	ΔG(1h1q)-ΔΔG(22→1h1q)
26	-8.43	-8.43 ± 0.47	0.00	ΔG(1h1q)-ΔΔG(26→1h1q)
28	-11.11	-9.50 ± 0.50	1.61	ΔG(1h1q)-ΔΔG(28→1h1q)
29	-9.88	-7.96 ± 0.56	1.92	ΔG(1h1q)-ΔΔG(29→1h1q)
30	-9.81	-8.48 ± 0.68	1.33	ΔG(1h1q)-ΔΔG(30→1h1q)
31	-9.54	-9.03 ± 0.19	0.51	ΔG(1h1q)-ΔΔG(31→1h1q)
**1h1q**	**-8.18**	**-8.18**	**0.00**	**experimental value**
1h1s	-11.25	-8.78 ± 0.60	2.47	ΔG(1h1q)-ΔΔG(1h1s→1h1q)
1oi9	-9.74	-9.18 ± 0.33	0.56	ΔG(1h1q)-ΔΔG(1oi9→1h1q)
1oiu	-9.08	-9.49 ± 0.26	0.41	ΔG(1h1q)-ΔΔG(1oiu→1h1q)
1oiy	-9.79	-8.68 ± 0.41	1.11	ΔG(1h1q)-ΔΔG(1oiy→1h1q)

[a] as reported by Wang et al. (2015)

Table S7. Experimental Δ G values with the corresponding calculated ΔG for the TYK2 system. The formula how ΔG was derived from ΔΔG by `Transformato` is given in the last column. The unsigned error (UE) is given for all values besides the reference structure.

	exp. ΔG^a	ΔG (TF)	UE (TF)	Calculation of Δ G
**ejm_31**	**-9.54**	**-9.54**	**0.00**	**experimental value**
ejm_42	-9.78	-10.07 ± 0.26	0.29	ΔG(ejm31)-ΔG(ejm42→ejm31)
ejm_43	-8.26	-6.66 ± 0.18	1.60	ΔG(ejm31)-ΔG(ejm43→ejm31)
ejm_44	-7.42	-5.45 ± 0.55	1.97	ΔG(ejm31)-ΔG(ejm44→ejm31)
ejm_45	-9.56	-9.91 ± 0.50	0.35	ΔG(ejm31)-ΔG(ejm45→ejm31)
ejm_46	-11.31	-8.74 ± 0.29	2.57	ΔG(ejm31)-ΔG(ejm46→ejm31)
ejm_47	-9.70	-9.06 ± 0.23	0.64	ΔG(ejm31)-ΔG(ejm47→ejm31)
ejm_48	-9.00	-7.94 ± 0.40	1.06	ΔG(ejm31)-ΔG(ejm48→ejm31)
ejm_49	-7.75	-7.58 ± 0.68	0.17	ΔG(ejm31)-ΔG(ejm49→ejm31)
ejm_50	-8.98	-10.26 ± 0.25	1.28	ΔG(ejm31)-ΔG(ejm50→ejm31)
ejm_54	-10.53	-9.29 ± 0.46	1.24	ΔG(ejm31)-ΔG(ejm54→ejm31)
ejm_55	-9.21	-9.68 ± 0.74	0.47	ΔG(ejm31)-ΔG(ejm55→ejm31)
jmc_23	-11.70	-9.19 ± 0.24	2.51	ΔG(ejm31)-ΔG(jmc23→ejm31)
jmc_28	-10.98	-7.46 ± 0.32	3.52	ΔG(ejm31)-ΔG(jmc28→ejm31)
jmc_30	-10.94	-8.04 ± 0.67	2.90	ΔG(ejm31)-ΔG(jmc30→ejm31)

[a] as reported by Wang et al. (2015)

Table S8. Distribution of absolute errors of calculated binding affinities with respect to experiment $\left|\Delta G^{bind}_{i,exp} - \Delta G^{bind}_{i}\right|$: Obs % is the percentage of deviations falling within the specified absolute error range. Anticip % is the percentage of deviations expected given an underlying Gaussian error distributions with RMSE of 1.17 kcal/mol (cf. Figure S7, dashed orange line).

absolute error (kcal/mol)	anticip %	obs %
<0.5	33.08	43.28
<1.0	60.72	64.18
<1.5	80.01	82.09
<2.0	91.26	91.04
<2.5	96.73	94.03
>2.5	3.26	5.97

REFERENCES

Gapsys, V., Pérez-Benito, L., Aldeghi, M., Seeliger, D., van Vlijmen, H., Tresadern, G., et al. (2020). Large scale relative protein ligand binding affinities using non-equilibrium alchemy. *Chem Sci* 11, 1140–1152. doi:10.1039/c9sc03754c

Homeyer, N. and Gohlke, H. (2013). FEW: A workflow tool for free energy calculations of ligand binding. *J Comput Chem* 34, 965–973. doi:10.1002/jcc.23218

Hu, Y., Sherborne, B., Lee, T. S., Case, D. A., York, D. M., and Guo, Z. (2016). The importance of protonation and tautomerization in relative binding affinity prediction: a comparison of AMBER TI and Schrödinger FEP. *J. Comput. Aided Mol. Des.* 30, 533–539. doi:10.1007/s10822-016-9920-5

Peterson, K. (2018). *Molecular basis for galectin-ligand interactions Design, synthesis and analysis of ligands*. Ph.D. thesis, Lund University, Faculty of Science, Department of Chemistry, Centre for Analysis and Synthesis

Wang, L., Wu, Y., Deng, Y., Kim, B., Pierce, L., Krilov, G., et al. (2015). Accurate and reliable prediction of relative ligand binding potency in prospective drug discovery by way of a modern free-energy calculation protocol and force field. *J. Am. Chem. Soc.* 137, 2695–2703. doi:10.1021/ja512751q
